# CLI-PPO: A Memory-Augmented PPO Framework with Intrinsic Curiosity and Curriculum Learning for Mapless Multi-Obstacle Navigation

**DOI:** 10.3390/s26196098

**Published:** 2026-09-25

**Authors:** Qifan Kang, Shigang Wang, Debing Xie

**Affiliations:** 1 School of Automation, Guangxi University of Science and Technology, Liuzhou 545006, China; 13753590430@163.com (Q.K.); xiedebing@yeah.net (D.X.); 2 Guangxi Low-Altitude Unmanned Aircraft Key Technologies Engineering Research Center, Liuzhou 545616, China

**Keywords:** mapless multi-obstacle autonomous navigation, deep reinforcement learning, CLI-PPO, curriculum learning, intrinsic curiosity module, Long Short-Term Memory

## Abstract

Mapless autonomous navigation in unknown multi-obstacle environments remains challenging for deep reinforcement learning due to limited perception, sparse rewards, and distribution shifts between training and deployment scenarios. Existing methods mainly rely on instantaneous observations or individual enhancement strategies, which often struggle to balance exploration robustness and navigation efficiency. This paper proposes a Curriculum Learning and Intrinsic Curiosity-enhanced Temporal Proximal Policy Optimization framework, referred to as CLI-PPO, for end-to-end autonomous navigation. The framework integrates temporal state representation, intrinsic exploration, and progressive task scheduling into PPO, where an LSTM network improves sequential decision-making under partial observability, an Intrinsic Curiosity Module enhances exploration in unfamiliar regions, and Curriculum Learning stabilizes policy optimization by gradually increasing task difficulty. A hybrid reward function combining target guidance, obstacle avoidance, and exploration incentives is designed for policy training. Experiments were conducted using ablation configurations, three independent training seeds, and an evaluation of one SAC policy under the corresponding task protocol, with 5000 randomized episodes considered at each obstacle-density level. CLI-PPO achieved a success rate of 66.24%, a collision rate of 33.58%, and an average return of 3433.14, compared with 46.06%, 53.94%, and 2083.21 for PPO, respectively. In the 10-obstacle setting, the three training seeds produced a CLI-PPO success rate of 62.72±3.59% and an average return of 3337.12±48.93, while PPO+ICM+Curriculum achieved 63.83±6.61% success and a slightly higher Euclidean-reference SPL. As obstacle density increased, PPO+ICM+Curriculum maintained higher success and SPL, whereas CLI-PPO yielded higher returns and more consistent performance across training runs.

## 1. Introduction

Autonomous navigation in an unknown environment with multiple obstacles requires a mobile robot to make continuous control decisions from incomplete local observations and limited task feedback. As robots move from structured industrial settings toward civilian and service applications, variations in obstacle configuration, sensing coverage, and free-space connectivity become increasingly important. In mapless navigation, the robot must infer feasible motion from local measurements and goal information without access to a precomputed global map.

Traditional path planning methods, such as A*, Dijkstra’s algorithm, Rapidly-exploring Random Tree, and their variants, rely heavily on optimization mechanisms that require prior global maps and accurate geometric information of the environment. In fully unknown scenarios or environments where robots rely solely on onboard LiDAR and low-dimensional sensors such as IMU, the inability to obtain global state information significantly reduces the generalization capability of these map-dependent approaches, making effective local path replanning difficult to achieve [1,2,3,4,5,6]. Therefore, under mapless navigation conditions without additional high-precision perception sensors, autonomous navigation in unknown multi-obstacle environments remains an important problem for mobile robots.

Deep reinforcement learning offers an alternative by learning a direct mapping from local observations and task feedback to continuous control commands. Representative methods include Q-learning, Deep Q-Networks, Soft Actor-Critic, Twin Delayed Deep Deterministic Policy Gradient, and Proximal Policy Optimization. These methods differ in how they reuse data, generate exploration, and update the policy, which affects their behavior in randomly generated environments with sparse goal feedback [7,8,9].

PPO is an on-policy policy-optimization algorithm based on a clipped surrogate objective. It collects rollouts with the current policy and limits the change between successive policy updates. This design makes the data distribution used for each update explicit and offers direct control over the update magnitude in continuous-control tasks [10].

SAC and TD3 are off-policy actor-critic algorithms that reuse transitions stored in a replay buffer. This can improve sample efficiency, but the buffer may contain data generated by earlier policies and different obstacle configurations. In randomly changing layouts, such a mixture may complicate critic estimation and safety-sensitive updates [11,12,13]. Their exploration mechanisms also differ. PPO samples from a stochastic policy, SAC adds entropy regularization, and TD3 generally relies on external exploration noise whose effect depends on the noise scale and task dynamics [14,15]. Off-policy methods have clear advantages and can outperform on-policy methods in suitable settings [16,17].

PPO is selected here for task-specific reasons. The navigation problem combines partial observability, collision-sensitive penalties, sparse goal events, and curriculum-induced changes in the task distribution. Current-policy rollouts and the clipped objective provide a controlled basis for combining recurrent state representation with intrinsic exploration. This task-dependent choice does not imply that PPO is generally superior to other continuous-control methods.

**Figure 1:**
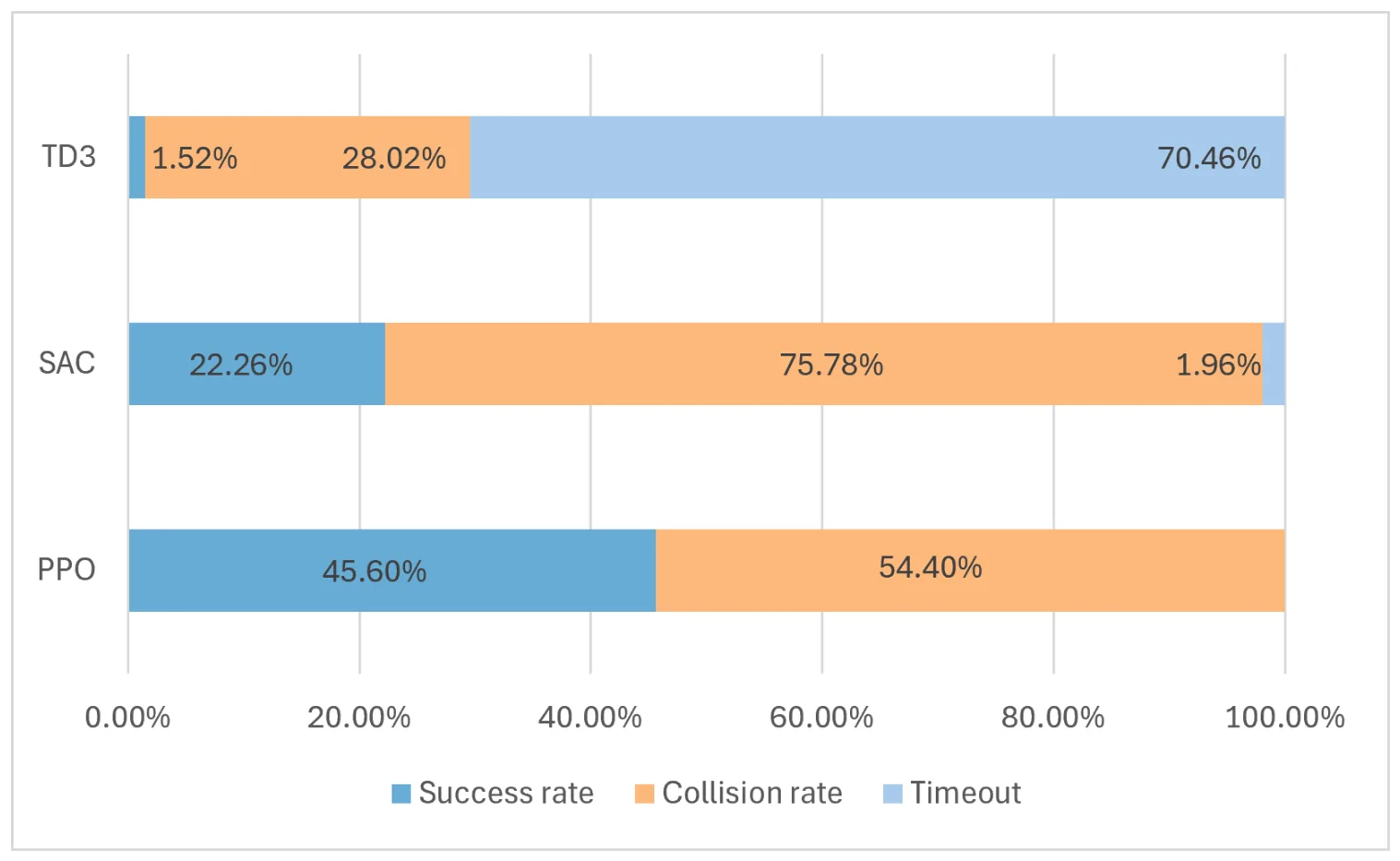
Percentage stacked bar chart comparing the success and collision rates of SAC, TD3, and PPO in the same-distribution testing environment based on 5000 random trials.

The preliminary comparison in Figure 1 evaluated PPO, SAC, and TD3 under the same task distribution. PPO achieved the highest success rate of 45.60%, while SAC reached 22.26% and exhibited a higher collision rate. TD3 achieved a success rate of only 1.52% and produced a timeout rate of 70.46%. Under this initial configuration, the evaluated PPO policy records the highest success rate and the lowest combined failure profile among the three reported policies.The deterministic off-policy updates and externally specified exploration noise of TD3 interact differently with the stochastic on-policy schedule and curriculum used in the present study, resulting in a training behavior that is not directly comparable with the PPO-based configurations.The subsequent quantitative analysis focuses on repeated training results for the PPO family and reports the behavior of one SAC policy under the corresponding task protocol.

Model-free reinforcement learning has also been investigated for decision-making problems in which an accurate analytical model is difficult to obtain. Valadbeigi et al. employed model-free Q-learning to achieve robust output consensus in heterogeneous multi-agent systems [18]. In robotic obstacle avoidance, Wang et al. developed an improved Soft Actor-Critic framework that incorporates local sensing, exploration, and safety-related costs [19]. Although this study also considers continuous control and obstacle avoidance, it is formulated for UUV dynamics and an off-policy optimization process, whereas the present work focuses on mapless indoor navigation based on local observations.

Other learning-based control studies also provide relevant methodological context. Lv et al. developed a nonlinear model-based control method for UAV motion regulation, emphasizing the role of dynamic feedback in continuous control [20]. Li et al. investigated adaptive feature representation for few-shot open-set recognition and its effect on generalization to unseen classes [21]. The former concerns feedback-based control under system dynamics, while the latter highlights the relationship between learned representations and generalization. CLI-PPO addresses these issues in navigation by encoding action-conditioned temporal sensor observations with a recurrent policy and evaluating the learned policy on unseen obstacle layouts.

Motivated by these considerations, CLI-PPO retains PPO as the optimization basis and integrates recurrent state representation, intrinsic curiosity, and curriculum learning. This design combines temporal context with guided exploration and progressive task scheduling for mapless navigation under partial observability and sparse goal feedback.

In this study, the robot uses LiDAR and onboard odometry or inertial information together with the relative goal state to make end-to-end navigation decisions. Local measurements do not provide a complete global description of the environment, so the navigation problem has three related difficulties. A single observation may not resolve perceptual aliasing [22]. Goal-reaching events are sparse over long episodes, which weakens purely reward-driven exploration [23]. Unconstrained local behavior can also lead to a safe but non-progressing policy [24].

Previous navigation studies have reported benefits from recurrent policies, intrinsic motivation, and curriculum learning, but they usually examine these strategies separately. Temporal models can improve decisions under partial observability, yet their history may become less informative as obstacle layouts change. Intrinsic rewards encourage exploration but can also favor task-irrelevant novelty. Curriculum learning stabilizes early training, although its progression must remain aligned with the agent capability. How these mechanisms interact in unknown multi-obstacle navigation therefore remains insufficiently studied.

To address these issues, we propose a curriculum-guided, curiosity-enhanced LSTM-PPO framework. The policy takes local LiDAR measurements, the target position relative to the robot, the angular difference between the robot heading and target direction, and the linear and angular velocities from the previous time step as inputs to generate end-to-end navigation commands in unknown multi-obstacle environments.

Within this framework, LSTM, ICM, and Curriculum Learning are organized around the three navigation challenges identified above. The recurrent policy integrates recent observations to reduce perceptual ambiguity, while ICM supplements sparse environmental feedback with an action-conditioned exploration signal. Curriculum Learning regulates the start–goal sampling range according to recent task performance, allowing the policy to encounter increasingly complex navigation conditions during training. These mechanisms interact through PPO optimization: recurrent features support policy and value estimation, intrinsic and extrinsic rewards jointly guide policy updates, and the curriculum determines the distribution of training tasks. Their integration therefore connects temporal representation, exploratory behavior, and progressive task adaptation within a unified navigation policy.

Specifically, the main contributions of this work are summarized as follows:

1. Incorporating a temporal memory network to enhance state representation. A Long Short-Term Memory network is embedded into the PPO policy, enabling the agent to infer contextual state information from historical observation sequences.This allows the policy to condition action selection on a sequence of observations in partially observable environments and under sensor perturbations;
2. Designing a boundary-range curriculum learning mechanism based on success-rate feedback. During the early training stage, the randomization range between the target point and the initial position is reduced to decrease task difficulty. As the agent’s success rate improves, the range is gradually expanded, effectively alleviating the difficulty of value function convergence in the initial stage and accelerating the smooth transition of the policy from simple to complex scenarios;
3. Integrating an intrinsic curiosity module to encourage efficient exploration. Beyond the extrinsic environmental reward, an intrinsic curiosity reward based on state-change prediction is introduced to motivate the agent to actively explore unknown regions. The Intrinsic Curiosity Module focuses more on action controllability and demonstrates greater stability in densely cluttered obstacle environments;
4. Reconstructing the reward function to overcome local optima. By enhancing goal-oriented attraction and introducing appropriate time penalties, a more goal-directed reward signal is constructed to guide the agent toward learning smooth and efficient navigation strategies. This effectively suppresses the local optimum behavior of being “safe but unable to make progress”;
5. Constructing unknown multi-obstacle environments in the MuJoCo physics simulation engine and systematically validating the proposed method. Additional simulation maps with different configurations from the training environments are developed for generalization evaluation.

The experimental results demonstrate that the proposed method significantly outperforms the standard PPO baseline in terms of both success rate and collision rate. Furthermore, ablation studies under the tested conditions and synergistic effectiveness of each component.

## 2. Related Work

This section reviews learning-based navigation under partial observability, sparse feedback, and changing task distributions, with emphasis on temporal representation, intrinsic exploration, and curriculum-based task scheduling.

### 2.1. Deep Reinforcement Learning and Its Applications to Path Planning

Mapless navigation requires a policy to generate continuous control commands from local observations and task-related information without access to a complete prior map. Deep reinforcement learning has been applied to unknown indoor environments, obstacle avoidance, multi-robot decision making, and aerial navigation [25,26,27,28,29]. These studies demonstrate the applicability of learned policies to continuous navigation, while also showing that their performance depends on partial observability, environmental variation, and the optimization procedure.

Recurrent PPO studies further emphasize the importance of sequence-aware training, hidden-state initialization, and episode-boundary handling [30,31]. These implementation issues are particularly relevant when local LiDAR observations are ambiguous and successive obstacle configurations are only partially observed.

### 2.2. Temporal Dependency and Generalization Challenges of Long Short-Term Memory Networks in Navigation Tasks

Recurrent policies retain information from previous observations when a single measurement does not fully describe the surrounding obstacle structure. LSTM has been widely used for sequential representation and has been extended through different recurrent architectures and feature-processing schemes [32,33,34,35,36,37,38]. Its usefulness in navigation depends on whether historical observations remain informative when obstacle number, size, or spatial arrangement changes [39]. The recurrent branch in the present study is therefore used to encode observation history under partial observability, while its effects on task completion and trajectory measurements are examined experimentally.

### 2.3. Curiosity Mechanisms for Exploration Enhancement and Their Inherent Limitations

Under sparse goal feedback, curiosity-based methods add an intrinsic signal related to the predictability or novelty of state transitions. The ICM formulation uses inverse and forward dynamics models in a learned feature space to obtain an action-conditioned prediction error [40]. Other studies have introduced curiosity through spiking networks, skill discovery, fuzzy exploration, and predictable sensorimotor representations [41,42,43,44]. Prediction error is not equivalent to navigation progress, because a difficult-to-predict transition may not help the robot approach the target or avoid obstacles. In the present framework, intrinsic feedback is therefore combined with the external navigation reward rather than treated as an independent navigation objective.

### 2.4. Curriculum Learning and the Challenges of Difficulty Mismatch and Catastrophic Forgetting

Curriculum learning changes the task distribution during training so that basic navigation behavior can be acquired before more demanding spatial configurations are introduced [45]. Related studies have examined automatic curriculum generation, task ordering, and adaptive task selection [46,47,48]. The timing of difficulty changes remains important because rapid expansion may expose the policy to configurations beyond its current capability. The curriculum used in this study adjusts the start–goal sampling boundary according to recent navigation success and gradually expands the training distribution.

These studies provide the methodological background for learning-based navigation under partial observability, sparse feedback, and changing task difficulty. The present work combines temporal observation processing, intrinsic exploration, and success-rate-based curriculum scheduling within a PPO-based navigation policy. Their measured effects are examined through component ablations, repeated training, obstacle-density variation, selected distribution-shift scenarios, and observation or control perturbations.

## 3. Experimental Evaluation and Results Analysis

This section describes the proposed CLI-PPO navigation framework and the simulation setting used to evaluate its different components. Because the obstacle layout is regenerated at the beginning of each episode, the training environment is represented in a structured form while retaining the main characteristics of mapless navigation, including local perception, partial observability, and stochastic obstacle distributions. This design makes it possible to examine the effects of temporal representation, intrinsic exploration, and curriculum learning under the same task conditions, while keeping the resulting navigation trajectories interpretable.

**Figure 2:**
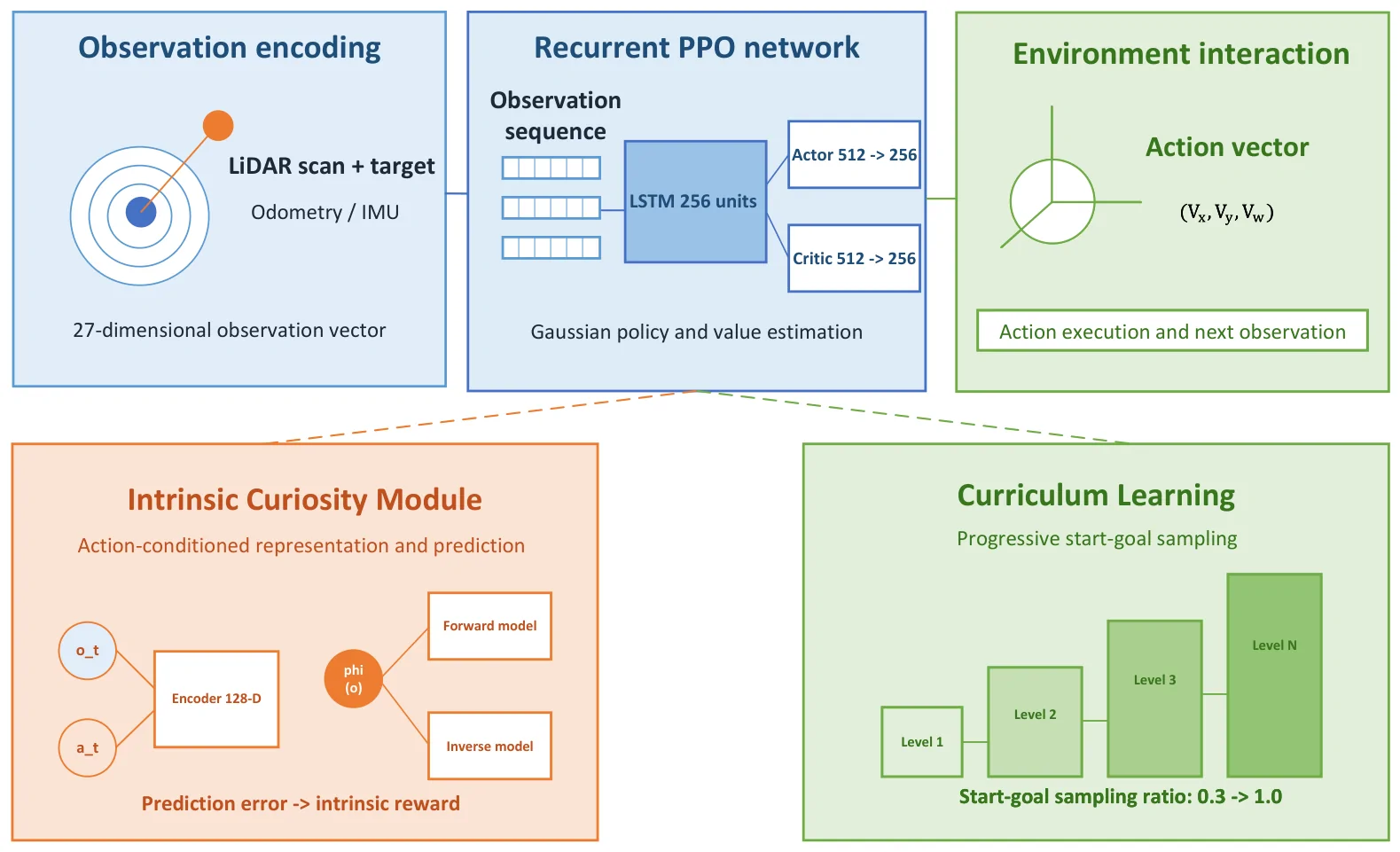
Overview of the CLI-PPO navigation framework. The 27-dimensional observation combines pooled LiDAR, target, angular, and motion information. An LSTM-based PPO network with Actor and Critic branches generates the continuous omnidirectional action $\left[v_{x},v_{y},\omega_{z}\right]$, while ICM provides action-conditioned intrinsic rewards and Curriculum Learning adjusts the start-goal sampling regions according to recent success feedback.

**Figure 3:**
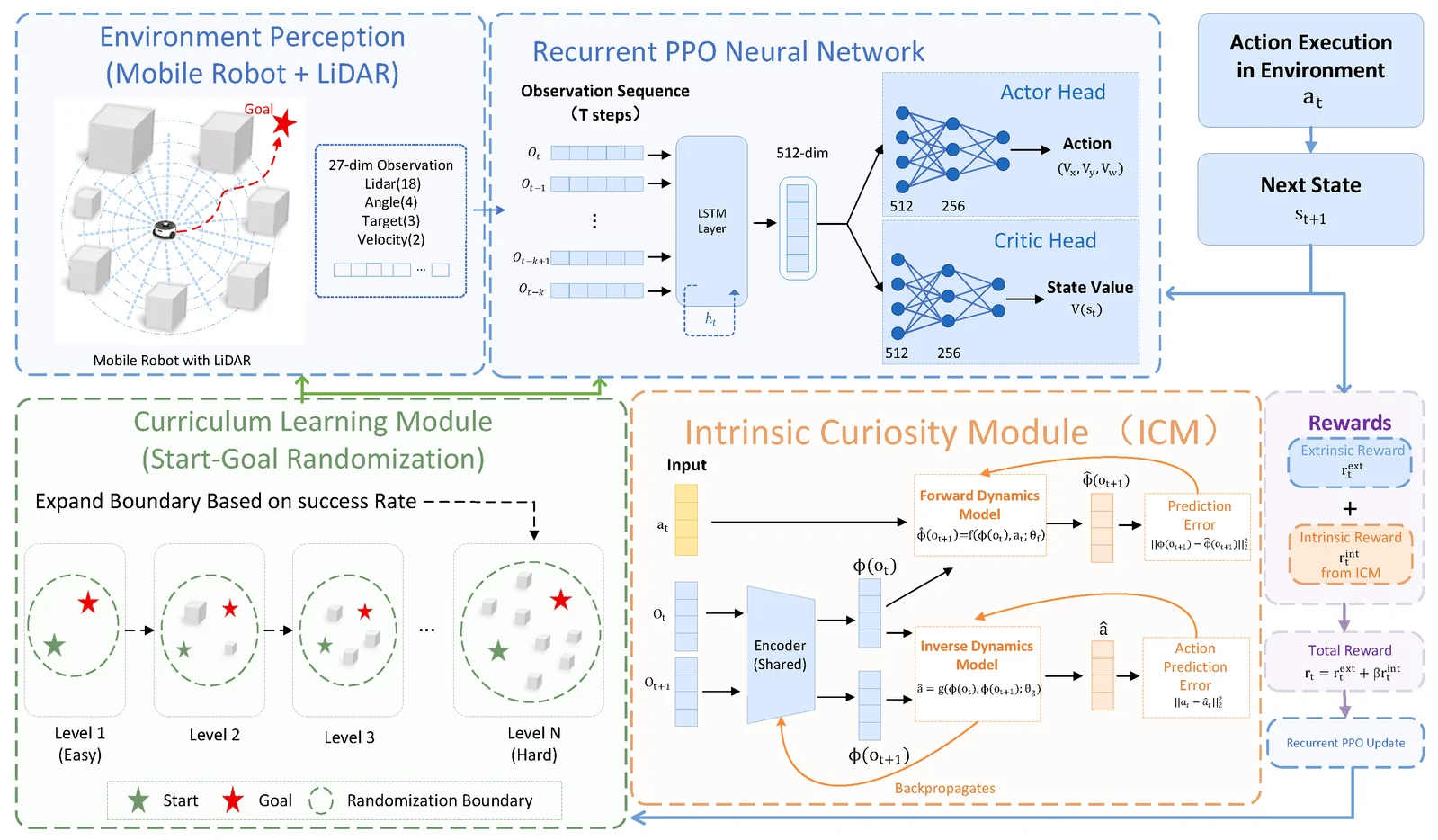
Overall training framework of CLI-PPO. The 27-dimensional observation is processed by the Recurrent PPO network for action generation and value estimation. Curriculum Learning adjusts task difficulty based on success-rate feedback, while ICM provides intrinsic rewards that are combined with extrinsic rewards for policy optimization.

An overview of the proposed framework is shown in Figure 2. The robot obtains local information from LiDAR, the relative target state, and onboard motion measurements. These observations are processed by the recurrent PPO policy, where the LSTM retains information from previous observations and the Actor and Critic branches generate continuous actions and estimate state values, respectively. The ICM uses the current observation and action to estimate the next state representation and provides an intrinsic reward based on the prediction error. At the same time, the curriculum mechanism adjusts the start-goal sampling range according to the recent navigation success rate. The detailed network structure, reward calculation, and interactions among these parts are given in Figure 3.

The navigation task is formulated as a Partially Observable Markov Decision Process. At each discrete time step *t*, the agent cannot access the global state of the environment and must make decisions from the local observation $O_{t}$. The observation is a 27-dimensional vector constructed from LiDAR, goal, and motion information. It includes the relative position between the robot and the target, denoted by dx and dy, the distance to the target, the sine and cosine encoding of the angular difference between the robot orientation and the target direction, the linear and angular velocities from the previous time step, and 18 distance values obtained by applying minimum pooling to 360 LiDAR rays.

Based on the kinematic constraints of the Omnidirectional Base, the low-level control action space is defined as a three-dimensional continuous vector, as shown in Equation (1).

(1) $$a_{t}={\left[v_{x},v_{y},\omega_{z}\right]}^{T}$$

where $v_{x}\in \left[-1,1\right]$ and $v_{y}\in \left[-1,1\right]$ represent the normalized longitudinal and lateral linear velocities of the mobile base in the local coordinate frame, respectively, while $\omega_{z}\in \left[-1,1\right]$ denotes the normalized angular velocity around the vertical axis. After receiving the action, the environment returns an immediate hybrid reward $r_{t}$ and evolves to the next state according to the transition probability distribution *P*. The primary optimization objective of the agent is to learn the optimal policy $\pi_{\theta }$ that maximizes the expected cumulative discounted return, as formulated in Equation (2).

(2) $$J\left(\theta \right)=E_{\pi_{\theta }}\left[\sum_{t=0}^{T}\gamma^{t}r_{t}\right]$$

where γ denotes the discount factor, and T=3000 represents the maximum exploration step length within a single episode.

### 3.1. Recurrent Proximal Policy Optimization with LSTM

Inspired by the human ability to learn from contextual information, this work addresses the state aliasing problem caused by single-frame LiDAR observations in Partially Observable Markov Decision Process scenarios. A shared Long Short-Term Memory layer is deployed before the feature extraction front-end of both the Actor and Critic networks. At an arbitrary time step t, the 27-dimensional observation sequence $o_{t}$, together with the previous hidden state $h_{t-1}\in R^{256}$ and cell state $c_{t-1}\in R^{256}$, is encoded by an LSTM layer with 256 hidden units to generate temporal hidden representations. The temporal information transition mechanism inside the LSTM can be formulated as Equation (3):

(3) $$h_{t},c_{t}=\mathrm{LSTM}\left(o_{t},h_{t-1},c_{t-1};\theta_{\mathrm{lstm}}\right)$$

where $h_{t-1}\in R^{256}$ represents a compact state representation that aggregates historical geometric boundaries and global motion trends.

During training, recurrent rollouts are collected from 24 parallel environments with a horizon of 2048 steps per environment. Instead of prescribing an additional fixed-length LSTM sequence, temporally consecutive samples from the same environment are organized into recurrent sequence segments. New segments are created at episode boundaries and at the boundaries between parallel environment trajectories, thereby preventing temporally unrelated transitions from being concatenated. The hidden and cell states stored at the beginning of each segment are used to initialize the corresponding recurrent computation. Within a minibatch, variable-length segments are padded to a common length, while a binary mask excludes padded positions from the policy, value, and entropy losses. The episode-start indicator resets the recurrent states when a new episode begins, preventing temporal information from propagating between independent navigation episodes. Policy optimization is performed with a nominal minibatch size of 128 over 10 epochs. Gradients are propagated through the valid transitions within each recurrent segment and truncated at its boundaries.

During evaluation, the recurrent state produced at the current time step is passed to the policy at the next time step and is reset when the corresponding episode terminates. This state-handling procedure preserves temporal context within an episode while avoiding information leakage across independently generated test scenarios.

Subsequently, the temporal features are separately fed into independent Multi-Layer Perceptrons of the Actor and Critic networks.The Actor and Critic branches use the same two-layer fully connected architecture, with a tanh activation applied between the hidden layers. The two branches then map the temporal representation to their respective outputs: the Actor produces the parameters of the continuous action distribution, whereas the Critic estimates the state value.

Taking the Actor network as an example, its deep nonlinear mapping process can be expressed as Equation (4):

(4) $$z_{t}^{\mathrm{actor}}=\tanh\left(W_{2}^{A}\;\tanh\left(W_{1}^{A}h_{t}+b_{1}^{A}\right)+b_{2}^{A}\right)$$

where the dimensions of the weight matrices are $W_{1}^{A}\in R^{512\times 256}$ and $W_{2}^{A}\in R^{256\times 512}$, respectively, and the bias vectors are $b_{1}^{A}\in R^{512}$ and $b_{2}^{A}\in R^{256}$. In this architecture, projecting the 256-dimensional temporal features into a 512-dimensional high-level latent space enables effective decoupling of highly entangled geometric topology and kinematic features in densely cluttered obstacle environments. The subsequent dimensionality reduction operation compresses the temporal features and reduces redundant information before policy prediction, forcing the network to filter out perception noise irrelevant to the current obstacle avoidance task.

Finally, the refined latent representation extracted through this deep mapping, $z_{t}^{\mathrm{actor}}\in R^{256}$, is linearly projected to parameterize the continuous policy distribution Equation (5):

(5) $$a_{t}∼N\left(\mu_{t},\sigma_{t}\right)$$

Here, $\mu_{t}$ and $\sigma_{t}$ denote the mean and standard deviation of the policy distribution, respectively, with the three action dimensions corresponding to the normalized velocity command $a_{t}={\left[v_{x},v_{y},\omega_{z}\right]}^{T}$. The components $v_{x}$ and $v_{y}$ represent the longitudinal and lateral linear velocities, while $\omega_{z}$ represents the angular velocity around the vertical axis. The policy learns the log-standard-deviation parameters jointly with the remaining policy parameters, so the exploration scale is not fixed during training.

At each interaction step, an action is sampled from the Gaussian distribution in Equation (5). Before execution, the three action components are constrained to the normalized interval [−1,1] through the control interface. The policy uses the unsquashed Gaussian parameterization; therefore, the action log-probability is evaluated directly under the Gaussian distribution used for sampling, without an additional change-of-variables correction. During evaluation, the mean of the policy distribution is used to generate a deterministic three-dimensional velocity command.

**Figure 4:**
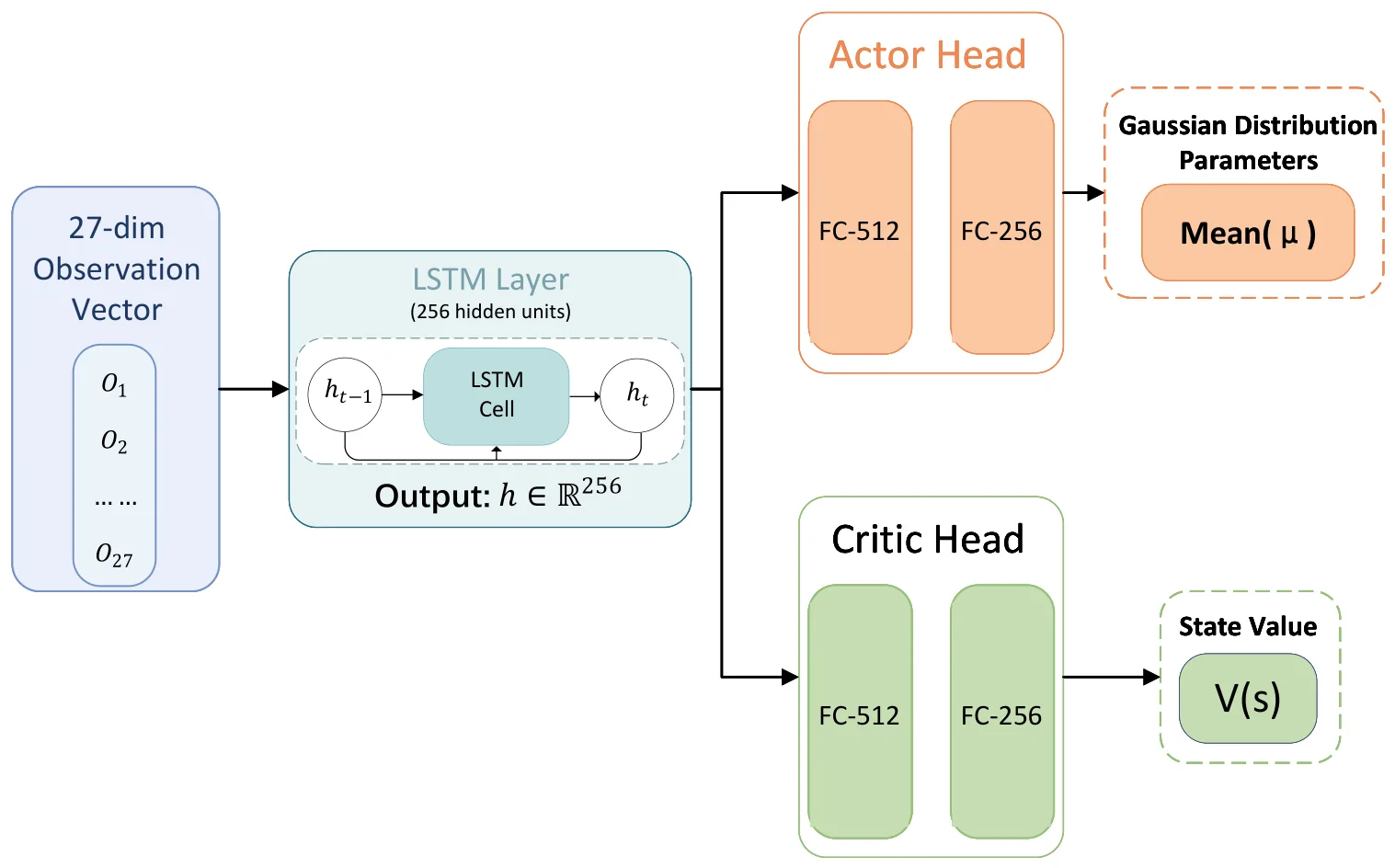
Architecture of the LSTM-based Actor-Critic network. The 27-dimensional observation vector is processed by an LSTM layer with 256 hidden units to extract temporal features. The hidden representation is shared by the Actor and Critic branches, where the Actor outputs Gaussian policy parameters through fully connected layers, and the Critic estimates the state value.

Similarly, the Critic branch performs parallel and symmetric tensor operations to output a scalar estimation of the current state value $V(s_{t})$. The resulting Actor–Critic architecture preserves the temporal information encoded by the LSTM and maps it to the policy and value outputs required for PPO optimization. The detailed architecture is illustrated in Figure 4.

### 3.2. Adaptive Curriculum Learning Based on Success-Rate Feedback

During long-horizon autonomous navigation training in indoor unknown densely cluttered obstacle environments, directly performing random sampling of the initial and target positions across the entire workspace at the early training stage results in an extremely low probability of the agent reaching the target region due to the inherent uncertainty of high-dimensional continuous action exploration. This severe sparsity of extrinsic reward feedback prevents the state-value estimation surface of the Critic Network from converging effectively over a prolonged period.

To overcome this cold-start bottleneck during the initial training phase, inspired by the progressive learning process from simple to complex tasks in human learning, this work develops an adaptive boundary-range curriculum learning mechanism based on empirical success-rate feedback. This mechanism enables the task difficulty to be dynamically adjusted according to the agent’s navigation performance, facilitating a gradual transition from simple scenarios to more challenging environments.

Although the integration of LSTM and ICM modules into the standard PPO framework can enhance the model’s representation capability and exploration efficiency, such architectural extensions inevitably increase the overall number of network parameters and the complexity of the optimization objectives. Since the standard PPO algorithm itself suffers from relatively high sample complexity and requires a large amount of interaction data to achieve effective policy fitting, the temporal dependencies introduced by LSTM and the auxiliary prediction task of ICM further intensify the optimization challenge. As a result, the network encounters more severe gradient instability and value function estimation bias during the early stage of training.

To address the optimization difficulty introduced by the expanded architecture, this study employs an adaptive curriculum learning mechanism based on success-rate feedback. The mechanism controls the rate at which task difficulty increases during training, allowing the policy to acquire navigation behavior under relatively accessible conditions before encountering more complex obstacle configurations.

The curriculum mechanism adjusts the task distribution by scaling the admissible sampling regions for the initial and target positions. At curriculum level *l*, these two spatial regions are regulated independently through the boundary variables $B_{\mathrm{start}}$ and $B_{\mathrm{goal}}$, as defined in Equations (6) and (7):

(6) $$B_{\mathrm{start}}=\alpha_{l}\cdot \left(B_{\mathrm{start}}^{\max}-B_{\mathrm{start}}^{\min}\right)+B_{\mathrm{start}}^{\min}$$

(7) $$B_{\mathrm{goal}}=\alpha_{l}\cdot \left(B_{\mathrm{goal}}^{\max}-B_{\mathrm{goal}}^{\min}\right)+B_{\mathrm{goal}}^{\min}$$

Here, $B_{\mathrm{start}}^{\min}$ and $B_{\mathrm{goal}}^{\min}$ denote the minimum admissible sampling ranges for the initial and target positions, respectively, while $B_{\mathrm{start}}^{\max}$ and $B_{\mathrm{goal}}^{\max}$ denote their corresponding maximum ranges. These boundary values are determined from the workspace geometry and the collision constraints before training. Because the two boundaries are scaled independently, Equations (6) and  (7) define a pair of spatial sampling regions rather than a single scalar constraint on the Euclidean distance between the initial and target positions. The feasible start–goal distance at a given curriculum level is jointly determined by the two sampling regions, workspace limits, obstacle-rejection conditions, and the minimum separation required by the environment.

The boundary ratio $\alpha_{l}$ is selected from the discrete set defined in Equation (8).

(8) $$\alpha_{l}\in \left\{\alpha_{0},\alpha_{1},\ldots ,\alpha_{l}\right\}$$

At the beginning of training $\alpha_{l}=\alpha_{0}$, and both the initial position and target position are constrained within a small region near the center of the environment. The current curriculum level is defined as l∈{0,1,…,L}, where the maximum number of curriculum stages is set to L = 10. The sampling boundary scaling coefficient $\alpha_{l}$ is generated through linear interpolation Equation (9):

(9) $$\alpha_{l}=\alpha_{\min}+\frac{l}{L}\left(\alpha_{\max}-\alpha_{\min}\right)$$

The initial value $\alpha_{\min}=0.3$ scales the initial and target sampling boundaries to a moderate portion of their respective predefined ranges while preserving variation in the start and goal configurations. As $\alpha_{l}$ increases toward $\alpha_{\max}=1.0$, these sampling regions gradually expand, exposing the policy to a broader range of spatial relationships and obstacle layouts. The curriculum thus provides a gradual transition from relatively accessible navigation conditions to the full predefined sampling ranges, without directly imposing a scalar Euclidean-distance constraint between the start and goal positions.

At the end of each environment episode, the system continuously maintains a sliding double-ended queue $B_{\mathrm{success}}$ with a capacity of N=50, which records the navigation outcomes of the latest 50 episodes. The adaptive curriculum advancement criterion is defined as Equation (10):

(10) $$l_{t+1}=\left\{\begin{array}{cc} l_{t}+1, & \mathrm{if}\;\frac{1}{N}\sum_{i=1}^{N}B_{i}\geq \rho \;\mathrm{and}\;l_{t}<L\\ l_{t}, & \mathrm{otherwise} \end{array}\right.$$

where ρ=0.7 denotes the success-rate threshold for curriculum promotion. The promotion decision is based on the outcomes stored in the sliding buffer $B_{\mathrm{success}}$, which contains the results of the most recent N=50 episodes. When the threshold is reached and the current level is below *L*, the curriculum advances to the next level and the corresponding sampling boundaries are expanded.

Following a curriculum transition, the expanded sampling boundaries remain unchanged for 100 interaction steps. The navigation outcomes collected during this interval are incorporated into $B_{\mathrm{success}}$, and the next curriculum update is determined from the resulting success-rate estimate. This procedure produces a gradual, performance-dependent expansion of the training task distribution.

The overall procedure of the Curriculum Learning mechanism is summarized in the pseudocode below Algorithm 1.
**Algorithm 1** Adaptive Curriculum Learning**Require:** Environment Env, success threshold ρ, buffer size *N*, curriculum levels *L***Ensure:** Curriculum level *l*, environment boundary Env.boundary  1:Initialize l←0, τ←0, B←∅  2:Set ρ←0.7, N←50, L←10, $\tau_{\max}\leftarrow 100$  3:α←linspace(0.3,1.0,L+1)  4:Env.boundary ←α[l]  5:**for** step=1:TotalTimesteps **do**  6:    **if** τ>0 **then**  7:        τ←τ−1  8:        Execute_PPO_Step()  9:        **continue**10:    **end if**11:    (done,is_success)← Execute_PPO_Step()12:    **if** done **then**13:        Add(B,is_success)14:    **end if**15:    **if** Size(B)≥N **then**16:        $R_{success}\leftarrow Mean\left(B\right)$17:        **if** $R_{success}\geq \rho$ **and** l<L **then**18:            l←l+119:            Env.boundary ←α[l]20:            Clear(B)21:            $\tau \leftarrow \tau_{\max}$22:        **end if**23:    **end if**24:**end for**25:**return** *l*, Env.boundary

### 3.3. Intrinsic Curiosity Module for Exploration Enhancement

In highly cluttered environments, Curriculum Learning can gradually expand the spatial sampling range, but it does not by itself resolve the exploration difficulty associated with local state transitions. When many actions lead to similar short-term outcomes, the policy may remain in a locally safe but ineffective behavior. This issue is particularly relevant when the external reward is sparse and collision penalties are large.

To provide an additional exploration signal, CLI-PPO incorporates an Intrinsic Curiosity Module (ICM) into the external navigation reward. The ICM is trained through self-supervised prediction of action-related state transitions. Rather than computing prediction errors directly in the raw observation space, the module first maps the observation $o_{t}\in R^{27}$ to a lower-dimensional latent representation. The 27-dimensional observation contains the motion-related and goal-related information together with the pooled LiDAR measurements. The ICM consists of a feature encoder ϕ, an inverse dynamics model *g*, and a forward dynamics model *f*.

The feature encoder ϕ is implemented as a two-layer multilayer perceptron with 256 hidden units. It maps the continuous observation $o_{t}$ to a 128-dimensional latent feature $\phi \left(o_{t}\right)\in R^{128}$. The inverse dynamics model uses the latent features of two consecutive observations to estimate the control action that produced the observed transition Equation (11):

(11) $${\hat{a}}_{t}=g\left(\phi \left(o_{t}\right),\phi \left(o_{t+1}\right);\theta_{g}\right)$$

where $\theta_{g}$ denotes the trainable parameters of the inverse dynamics model. By optimizing the inverse prediction objective, the feature encoder is encouraged to extract action-relevant dynamic information while filtering out environmental variations unrelated to the agent’s controllable motion.

The optimization objective of the inverse dynamics model is to minimize the Mean Squared Error (MSE) between the inferred action and the actual omnidirectional control action $a_{t}\in R^{3}$ Equation (12):

(12) $$L_{\mathrm{inv}}=\frac{1}{3}\sum_{i=1}^{3}{\left({\hat{a}}_{t,i}-a_{t,i}\right)}^{2}$$

where ${\hat{a}}_{t,i}$ and $a_{t,i}$ denote the *i*-th dimension of the predicted action and the actual control action, respectively. Minimizing this loss encourages the latent representation extracted by the feature encoder to preserve the dynamic information that is directly related to the agent’s controllable state transitions.

Based on the latent feature representation “purified” by the inverse dynamics model, the Forward Dynamics Model f takes the current latent state representation $\phi (o_{t})$ and the actual action $a_{t}$ executed by the agent as inputs, aiming to predict the latent feature representation of the next observation Equation (13):

(13) $$\hat{\phi }\left(o_{t+1}\right)=f\left(\phi \left(o_{t}\right),a_{t};\theta_{f}\right)$$

where $\theta_{f}$ denotes the trainable parameters of the forward dynamics model. By learning the latent transition dynamics induced by the agent’s actions, the forward model captures the predictability of environmental state evolution and provides the basis for constructing the intrinsic curiosity reward.

The core principle of the Intrinsic Curiosity Module is that a larger prediction error produced by the forward model for a specific state transition indicates that the transition is more novel and contains more informative content for the current network. Therefore, the single-step intrinsic curiosity reward $r_{t}^{\mathrm{int}}$ is rigorously defined as the mean squared error of the forward feature prediction error Equation (14):

(14) $$r_{t}^{\mathrm{int}}=\frac{1}{128}{∥\hat{\phi }\left(o_{t+1}\right)-\phi \left(o_{t+1}\right)∥}_{2}^{2}$$

where $\hat{\phi }\left(o_{t+1}\right)$ denotes the predicted latent feature representation generated by the forward dynamics model, and $\phi (o_{t+1})$ represents the actual latent feature representation encoded from the next observation. The normalization factor $\frac{1}{128}$ corresponds to the dimensionality of the latent feature space, ensuring that the intrinsic reward magnitude remains stable and independent of the feature dimension.

By maximizing this prediction-error-based intrinsic reward, the agent is encouraged to explore state transitions that are difficult to predict under the current dynamics model, thereby improving exploration efficiency in unknown environments while avoiding excessive dependence on sparse extrinsic navigation rewards.

The loss function of the Forward Dynamics Model $L_{\mathrm{fwd}}$ is defined as the expected prediction error over the entire training batch Equation (15):

(15) $$L_{\mathrm{fwd}}=\frac{1}{N}\sum_{i=1}^{N}\frac{1}{128}{∥\hat{\phi }\left(o_{t+1}^{i}\right)-\phi \left(o_{t+1}^{i}\right)∥}_{2}^{2}$$

where *N* denotes the batch size. During each gradient update stage, the parameters of the feature encoder, forward dynamics model, and inverse dynamics model $\left(\theta_{\phi },\theta_{f},\theta_{g}\right)$ are optimized through independent joint backpropagation, which is decoupled from the main PPO policy network.

The overall self-supervised training objective of the ICM module is to minimize the unweighted linear combination of the inverse dynamics loss and the forward dynamics loss, as defined in Equation (16):

(16) $$\min_{\theta_{\phi },\;\theta_{f},\;\theta_{g}}L_{\mathrm{icm}}=L_{\mathrm{fwd}}+L_{\mathrm{inv}}$$

The two terms in Equation (16) are assigned unit coefficients. This choice provides a simple joint-training objective without introducing an additional loss-balancing parameter. As indicated by Equations (12) and (15), the inverse and forward errors are averaged over the action dimensions and the 128-dimensional latent feature, respectively. These averaging operations define the normalization convention used by the implementation. They do not imply that the two losses have identical numerical values or identical gradient magnitudes.

The intrinsic reward is combined with the complete external navigation reward according to

(17) $$r_{t}=r_{t}^{\mathrm{ext}}+\beta r_{t}^{\mathrm{int}},$$

where $r_{t}^{\mathrm{ext}}$ denotes the complete task reward defined in the reward function, including goal-achievement reward, distance and heading shaping terms, collision penalties, and time-related terms. The coefficient β controls the relative contribution of the prediction-based intrinsic reward to the policy optimization process. Because the external reward contains terms with substantially different magnitudes, changing β can alter the balance between task completion and exploratory behavior.

To examine the effect of the intrinsic-reward coefficient during policy optimization, three CLI-PPO policies were trained separately with fixed values of β∈{0.05,0.10,0.20}. The network architecture, environment configuration, curriculum settings, training procedure, and all other reward terms were kept unchanged across the three training runs. After each training run, the resulting policy parameters and observation-normalization statistics were fixed for evaluation. Each policy was evaluated using deterministic actions over the same 5000-episode scenario set. Therefore, the results in Table 1 describe the navigation behavior of three policies learned under different training coefficients; β was not changed during policy evaluation.

The coefficient β=0.05 achieves a success rate of 54.8%, with a collision rate of 44.4% and an average return of 2850.23. Increasing the coefficient to β=0.10 improves the success rate to 65.6% and reduces the collision rate to 34.2%. The average return also increases to 3384.57, while the Euclidean-reference SPL improves from 0.460 to 0.565. These results indicate that a moderate intrinsic-reward contribution can provide a more effective exploration signal in the presence of sparse goal rewards and collision-sensitive navigation objectives.

When β is further increased to 0.20, the success rate decreases slightly to 63.8%, and the average return decreases to 3286.03. The Euclidean-reference SPL also decreases slightly to 0.558, although the collision and timeout rates remain comparable to those obtained with β=0.10. This pattern is consistent with diminishing returns when the intrinsic reward is given excessive weight: stronger exploration incentives do not necessarily produce better task completion or trajectory efficiency.

Overall, β=0.10 provides the best empirical trade-off among success rate, collision avoidance, cumulative return, and trajectory efficiency within the tested range. However, the confidence intervals of the tested coefficients overlap, particularly between β=0.10 and β=0.20. Therefore, these results demonstrate a coefficient-dependent performance trend rather than proving that β=0.10 is universally optimal. The selected value is retained as an empirically effective setting for the CLI-PPO configuration considered in this study.

### 3.4. Environment Modeling

In mobile robot autonomous navigation environment modeling, it is essential to simultaneously consider the real-time computational efficiency of simulation and the generality of task scenarios. Although traditional discrete grid-based or topological graph representations are simple to construct and computationally efficient for planning, they are insufficient for accurately characterizing the continuous motion dynamics of robots and the detailed sensor feedback from devices such as LiDAR in unknown densely cluttered environments.

**Figure 5:**
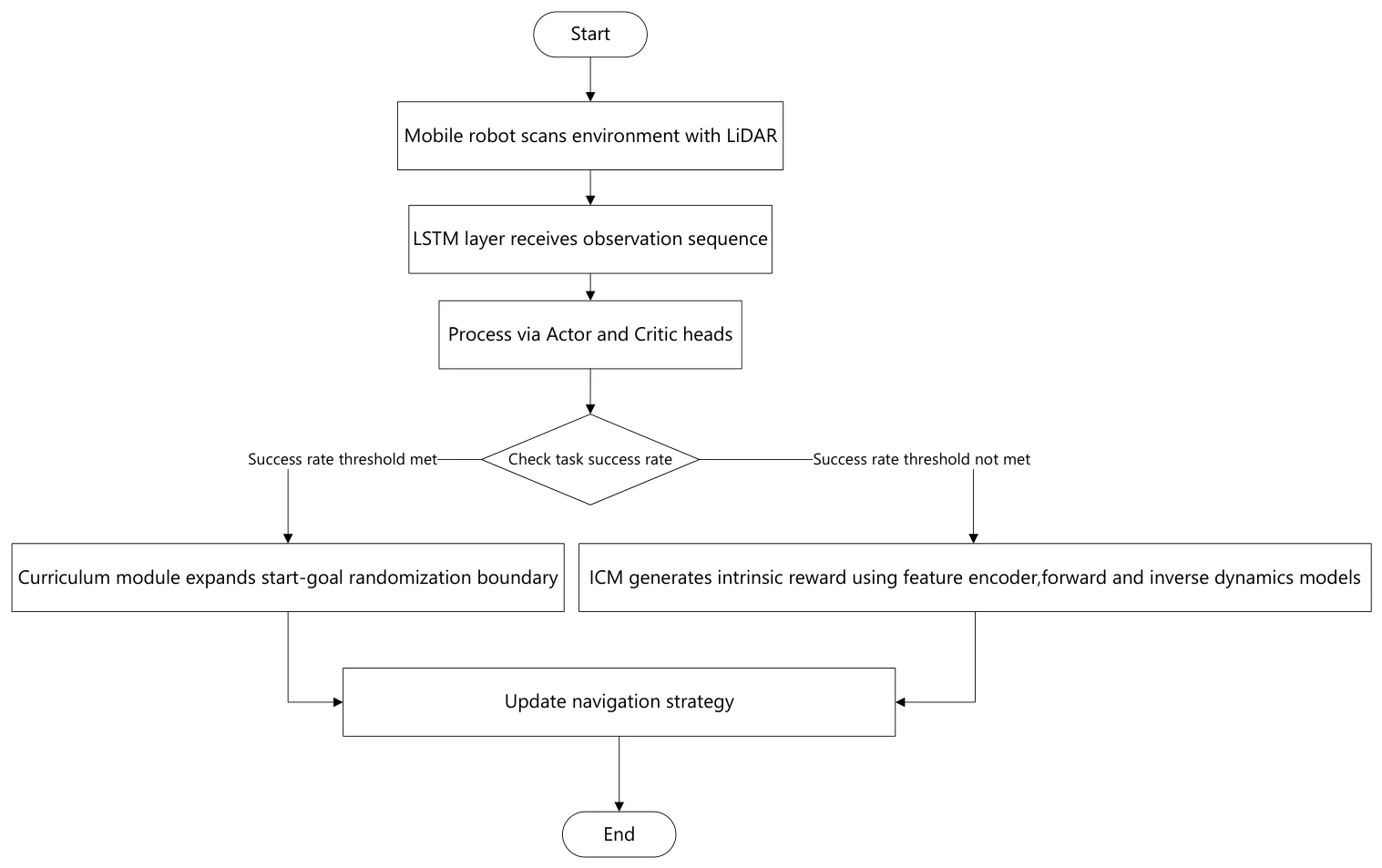
Training procedure of the proposed CLI-PPO framework. The agent obtains LiDAR observations, extracts temporal features through LSTM, and optimizes the policy using Actor-Critic networks. Curriculum Learning adjusts task difficulty based on success rate, while ICM provides intrinsic rewards through feature prediction to guide exploration.

To obtain high-fidelity interaction data, this paper employs a physics engine to construct a continuous-space two-dimensional navigation simulation environment. Furthermore, a deployment-compatible strategy is designed under the framework of the open-source robotic middleware ROS2, enabling seamless integration of the proposed navigation policy into robotic systems. The overall workflow is illustrated in Figure 5.

The simulation environment is configured as a closed rectangular area with dimensions of $8\times 8\;m^{2}$, surrounded by walls with a fixed height, where the internal region represents the robot’s feasible operating space. The obstacle configurations are divided into two stages: training and evaluation.

During training, 10 static obstacles are randomly generated within the environment. The shapes of these obstacles are randomly selected as either cylinders or cuboids, with their dimensions uniformly sampled within the range of 0.1m to 0.25m. In each episode, both the initial position and the target position are reset.

It should be noted that, compared with real-world indoor multi-obstacle environments, the simulated maps constructed in this study are simplified in terms of obstacle density, geometric complexity, and environmental dynamics. This simplification is not intended to disregard the complexity of real-world scenarios, but rather to provide a controlled and reproducible platform for evaluating the proposed framework. The primary motivation is to systematically analyze the individual contributions and interactions of the Curriculum Learning, Intrinsic Curiosity Module, and temporal memory network components. By reducing the interference introduced by excessive environmental complexity, the effects of different modules can be more clearly identified through ablation studies. Moreover, training reinforcement learning policies in highly complex environments generally requires significantly more interaction samples and computational resources due to the increased difficulty of exploration and policy convergence. Therefore, a simplified yet representative environment is adopted to enable fair comparisons among multiple algorithm variants while maintaining the essential characteristics of the navigation task.

Based on this fundamental environment, this work primarily focuses on validating the proposed algorithmic framework and improving the interpretability of the methodology. This provides a solid theoretical baseline for future extensions toward denser, more dynamic, and realistic multi-obstacle navigation scenarios.

The robot adopts an omnidirectional mobile base model, with its collision geometry simplified as a cylinder with a radius of 0.15 m. The perception system consists of a two-dimensional LiDAR sensor with a $360^{\circ }$ scanning range. Each laser beam continuously measures the distance from the robot to obstacles along its corresponding direction, providing local depth information of the surrounding environment.

Since the LiDAR sensor can only perceive the local field of view and the global map of the environment is completely unavailable, the navigation task naturally exhibits the characteristics of a Partially Observable Markov Decision Process (POMDP).

Accordingly, the raw sensor information is constructed into a 27-dimensional observation vector $o_{t}$, which consists of the following components:

- The two-dimensional relative displacement $\left(d_{x},d_{y}\right)$ between the target point and the robot’s current position, along with the corresponding Euclidean distance;
- The sine and cosine encoding of the robot’s current heading angle;
- The sine and cosine encoding of the relative bearing angle between the robot and the target;
- The previous time step’s planar velocity feedback, including the longitudinal and lateral velocity components, which provides dynamic motion context for the decision-making network;
- The 18-dimensional minimum depth values extracted from $360^{\circ }$ LiDAR rays through ray grouping and Min-pooling, which are used to characterize the local obstacle topology surrounding the robot in detail.

On the control side, the action space is defined as a three-dimensional continuous vector ${\left[v_{x},\;v_{y},\;\omega_{z}\right]}^{T}$ where $v_{x}$ and $v_{y}$ represent the normalized desired longitudinal and lateral linear velocities, respectively, and $\omega_{z}$ denotes the normalized desired angular velocity. These control commands are linearly scaled at the low-level control layer and converted into the robot’s actual velocity commands within the physics engine, according to the velocity limits of the simulated platform.

To guide the agent to efficiently approach the target while avoiding obstacles, the environment provides a hybrid reward function composed of a dense progress-based guidance term and sparse event-based terms. Based on the predefined hyperparameters, the single-step extrinsic reward $r_{t}^{\mathrm{ext}}$ is as Equation (18):

(18) $$r_{t}^{\mathrm{ext}}=r_{d}\cdot \Delta d_{\mathrm{approach}}+r_{h}\cdot \Delta h_{\mathrm{approach}}+r_{g}\cdot I_{\mathrm{goal}}+r_{c}\cdot I_{\mathrm{collision}}+r_{t}$$

where the distance improvement term toward the target and the heading improvement term are defined as Equations (19) and (20):

(19) $$\Delta d_{\mathrm{approach}}=\max\left(d_{t-1}-d_{t},\;0\right)$$

(20) $$\Delta h_{\mathrm{approach}}=\max\left(|\psi_{t-1}|-|\psi_{t}|,\;0\right)$$

Here, $\Delta d_{\mathrm{approach}}$ represents the reduction in Euclidean distance between the robot and the target, while $\Delta h_{\mathrm{approach}}$ denotes the improvement in the absolute heading deviation toward the target. $I_{\mathrm{goal}}$ and $I_{\mathrm{collision}}$ are binary indicator functions representing successful target arrival and collision with obstacles, respectively. The term $r_{t}$ represents a constant time penalty applied at each step, which encourages the robot to minimize unnecessary interactions and avoid inefficient wandering behaviors.

Table 2 summarizes symbols involved in the reward function, including the empirically defined coefficients, state-dependent reward components, and the corresponding intermediate variables.

An episode is terminated when any of the following conditions is satisfied:

- The Euclidean distance between the robot center and the target point is smaller than the threshold $\lambda_{\mathrm{agent}}$, which is considered a successful arrival at the target;
- The minimum LiDAR measurement is lower than the collision threshold $\lambda_{\mathrm{agent}}+0.05\;m$, indicating that a collision has occurred;
- The number of interaction steps reaches the maximum limit T = 3000, which is regarded as a timeout.

In addition, all observations and reward signals are processed through online normalization using VecNormalize during training to improve numerical stability and facilitate more stable neural network optimization.

### 3.5. Training Setup and Procedure

In the omnidirectional mobile robot simulation environment constructed above, this section provides a detailed description of the hardware configuration, training settings, baseline comparison schemes, and the complete end-to-end training procedure of the proposed algorithm. All training and evaluation experiments were conducted on a high-performance computing server equipped with an NVIDIA RTX 5090 GPU and an Intel Core i9-14900KF. To accelerate experience collection in the high-dimensional continuous action space, the training environment was parallelized using multiple independent subprocess environments.

The network parameters were optimized using the Adam optimizer, and the proposed framework was trained with a fixed number of environment interaction steps. The reinforcement learning training process adopted the standard PPO optimization paradigm, where the policy update was constrained by the trust-region clipping mechanism. To improve training stability, all local observation features and hybrid reward signals were normalized using online running statistics through VecNormalize. The Intrinsic Curiosity Module (ICM) was implemented as an auxiliary branch and jointly optimized with the main PPO policy network through synchronous backpropagation.

The detailed hyperparameter configurations of the proposed algorithm are summarized in Table 3.

To rigorously evaluate the effectiveness of each key component in the proposed CLI-PPO framework, a series of progressively augmented ablation baselines were designed under the same hybrid reward function and identical training timesteps. The comparative baselines are defined as follows:

- PPO: The standard Proximal Policy Optimization baseline without any temporal memory or exploration enhancement mechanisms;
- PPO+LSTM: Incorporates a recurrent hidden layer to provide a fundamental memory-based baseline for addressing the partially observable nature of the POMDP environment;
- PPO+ICM: Extends the standard PPO framework with an Intrinsic Curiosity Module to enhance exploration efficiency through intrinsic motivation;
- PPO+LSTM+ICM: Combines temporal feature encoding and curiosity-driven exploration mechanisms to improve state representation and exploration capability;
- PPO+ICM+Curriculum: Integrates intrinsic motivation with dynamic curriculum-based task scheduling to facilitate progressive policy learning;
- CLI-PPO: The complete proposed framework, which jointly integrates LSTM-based temporal representation, ICM-driven exploration, and Curriculum Learning-based adaptive task scheduling.

**Figure 6:**
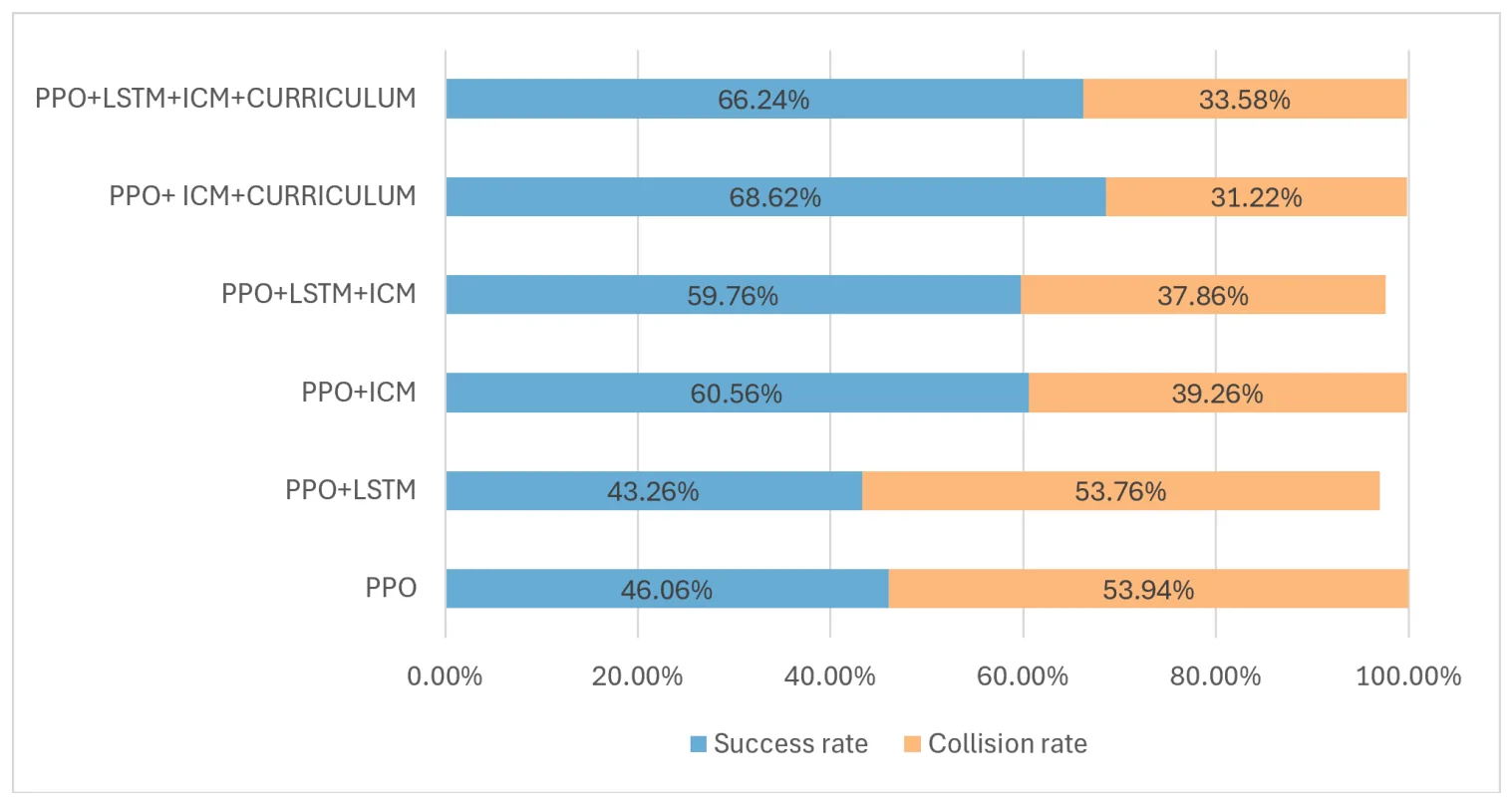
Success rate and collision rate of six algorithm configurations under the in-distribution testing environment.

**Figure 7:**
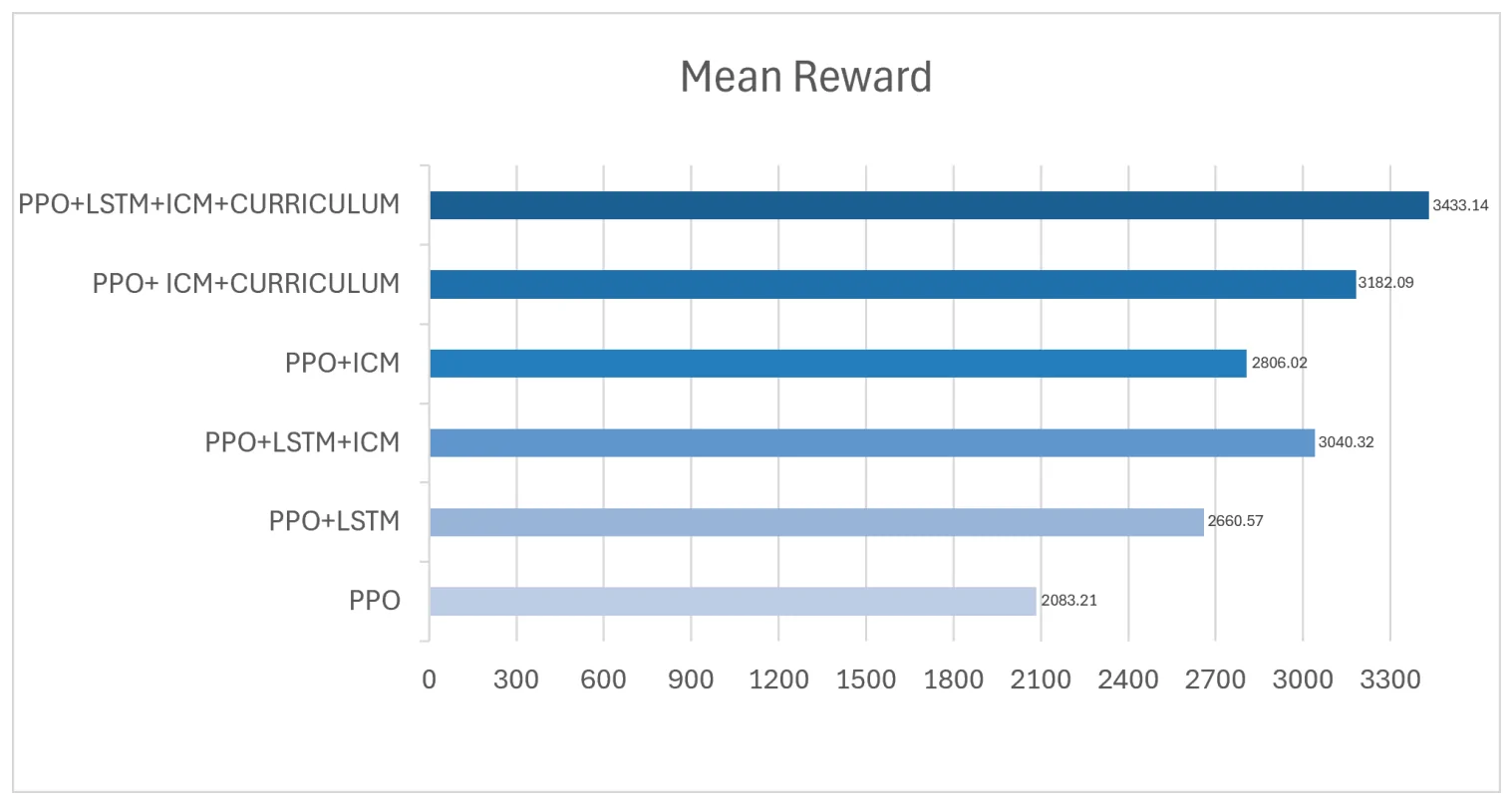
Comparison of average episodic returns among six algorithm variants under the in-distribution testing environment.

The six configurations described above constitute the original ablation study, and their in-distribution results are presented in Figure 6 and Figure 7. These results provide the primary comparison of the component combinations under the original evaluation setting.

To further characterize the variability associated with policy training, PPO, PPO+LSTM, PPO+ICM+Curriculum, and CLI-PPO were independently trained with random seeds 43, 44, and 2026, while the remaining training settings followed Table 3. Each independently trained policy was evaluated for 5000 randomized episodes under four obstacle-density conditions containing 10, 15, 20, and 25 obstacles. The Euclidean distance between the initial and target positions was maintained above 4 m, and the same scenario-generation procedure was applied to all compared policies. Intrinsic curiosity rewards were disabled during evaluation, and the curriculum boundary restrictions used during training were removed.

For each policy and obstacle-density condition, the episode-level metrics were first averaged within each training seed. The reported PPO-family values were then calculated as the arithmetic mean and sample standard deviation of the three seed-level means. The evaluation included success rate, collision rate, timeout rate, cumulative return, traveled path length, normalized path length, Euclidean-reference SPL, minimum obstacle clearance, and trajectory smoothness. This aggregation provides a seed-level description of training variability in addition to the original ablation results.

One SAC policy was evaluated under the same task definition, observation representation, reward formulation, obstacle-density conditions, episode limit, and 5000-episode testing protocol. Its replay-buffer and entropy-regularized updates remained specific to the SAC algorithm. The SAC values represent the mean performance obtained over 5000 evaluation episodes for each obstacle-density condition.

The complete training pseudocode is presented as follows Algorithm 2.
**Algorithm 2** CLI-PPO End-to-End Training Framework  1:Initialize PPO policy parameters θ, LSTM parameters $\theta_{lstm}$, ICM parameters $\theta_{ICM}=\left\{\theta_{\phi },\theta_{f},\theta_{g}\right\}$, rollout buffer B and success buffer $B_{success}$  2:Set $T_{max}=5\times 10^{7}$, N=24, $n_{steps}=2048$  3:Set learning rate $lr=1\times 10^{-5}$, discount factor γ=0.999, intrinsic reward coefficient β=0.1, and curriculum threshold ρ=0.7  4:Initialize curriculum boundary ratio α=0.3  5:**for** iter=1 to $\frac{T_{max}}{N\cdot n_{steps}}$ **do**  6:    **for** step=1 to $n_{steps}$ **do**  7:        **for** i=1 to *N* **do**  8:            Obtain observation: $o_{t}^{i}\leftarrow \mathrm{GetObservation}\left(Env_{i}\right)$  9:            Update recurrent hidden states: $h_{t}^{i},c_{t}^{i}\leftarrow \mathrm{LSTM}\left(o_{t}^{i},h_{t-1}^{i},c_{t-1}^{i};\theta_{lstm}\right)$10:            Sample continuous action: $a_{t}^{i}∼\pi_{\theta }\left(a_{t}^{i}|h_{t}^{i}\right)$11:            Execute action: $\left(o_{t+1}^{i},r_{ext}^{i},done^{i}\right)\leftarrow \mathrm{Step}\left(Env_{i},a_{t}^{i}\right)$12:            Compute intrinsic curiosity reward: $r_{int}^{i}\leftarrow \mathrm{ComputeICM}\left(o_{t}^{i},o_{t+1}^{i},a_{t}^{i};\theta_{ICM}\right)$13:            Compute total reward: $r_{t}^{i}=r_{ext}^{i}+\beta r_{int}^{i}$14:            Store transition: $B\leftarrow (o_{t}^{i},h_{t}^{i},a_{t}^{i},r_{t}^{i},o_{t+1}^{i})$15:        **end for**16:    **end for**17:    Compute ICM loss: $L_{ICM}\leftarrow \mathrm{ComputeICMLoss}\left(B\right)$18:    Update ICM parameters: $\theta_{ICM}\leftarrow \mathrm{AdamUpdate}\left(\theta_{ICM},L_{ICM}\right)$19:    Update PPO policy: $\left(\theta ,\theta_{lstm}\right)\leftarrow \mathrm{PPOUpdate}\left(\theta ,\theta_{lstm},B\right)$20:    Update success buffer: $B_{success}\leftarrow \mathrm{UpdateSuccessBuffer}\left(done\right)$21:    **if** $\mathrm{Mean}(B_{success})\geq \rho$ **then**22:        Expand curriculum boundary: α←ExpandBoundary(α)23:        Update environment boundary: SetBoundary(Env,α)24:    **end if**25:    Clear rollout buffer: B←∅26:**end for**27:**return** $\theta ,\theta_{lstm}$

### 3.6. Experimental Results

Based on the ablation results presented in Figure 6 and Figure 7, the influence of different modules on the navigation performance of the agent can be further analyzed. The standard PPO method achieves only a 46.06% success rate in the testing environment, with a collision rate of 53.94%, suggesting that a purely reactive policy based on single-step observations may be less effective in handling the spatial constraints of complex obstacle environments. During training, due to the lack of continuous modeling of historical states, the agent tends to make myopic decisions based on current local observations. When obstacle configurations become complex or feasible paths are highly constrained, the policy struggles to establish stable obstacle avoidance behaviors, resulting in a high failure probability.

After introducing the LSTM module, the average return increases from 2083.21 to 2660.57, whereas the success rate decreases from 46.06% to 43.26%. The recurrent policy therefore changes the reward accumulation pattern without increasing the proportion of successfully completed episodes under the present training configuration. Historical observations are incorporated into the policy state, but temporal representation alone does not provide the additional exploration signal required by the sparse-reward navigation task. The effect of the recurrent branch is consequently examined through its use of sequential observations and its interaction with the exploration and curriculum mechanisms.

In contrast, incorporating the ICM module increases the success rate to 60.56% and reduces the collision rate to 39.26%, compared with 46.06% and 53.94% for PPO, respectively. The average return also increases to 2806.02. This performance pattern suggests that the prediction-error signal provides additional transition-level guidance when extrinsic navigation feedback is sparse. By supplementing the task reward with information about the predictability of recent state changes, ICM encourages the policy to explore a broader range of state transitions, which is associated with improved task completion under the present training conditions.

Adding Curriculum Learning further increases the success rate to 68.62% and reduces the collision rate to 31.22%. The additional improvement indicates that exploration guidance alone does not fully address the difficulty of learning across increasingly complex task configurations. ICM supplies transition-level exploratory feedback, whereas Curriculum Learning regulates the distribution of start–goal configurations and gradually expands the spatial sampling range. Without this progressive task scheduling, the policy must learn exploratory behavior and obstacle-avoidance decisions over a highly variable task distribution from the beginning, which can make value estimation and policy optimization more difficult. The curriculum first exposes the policy to relatively accessible configurations and then broadens the range of obstacle layouts and spatial relationships. In this way, the two mechanisms contribute to different stages of the learning process: ICM enriches exploration, while Curriculum Learning controls the progression of task difficulty.

It is worth noting that although PPO+LSTM+ICM simultaneously integrates temporal memory and exploration mechanisms, its performance remains inferior to the model equipped with curriculum learning, achieving only a 59.76% success rate and an average reward of 3040.32. This phenomenon indicates that increasing network complexity does not necessarily guarantee performance improvement. LSTM enhances historical state representation, while ICM increases the tendency to explore unknown regions; however, their combined effects also enlarge the policy optimization space, requiring the agent to simultaneously learn temporal representation, exploration behaviors, and adaptation to complex environments. Without curriculum guidance, the training process is more susceptible to complex state distributions and exploration noise, leading to unstable value estimation and limiting further policy improvement. Therefore, curriculum learning is not merely a mechanism for expanding exploration space; rather, it provides a progressive capability transfer process from simple to complex tasks, allowing the LSTM and ICM modules to function under more stable optimization conditions.

The complete CLI-PPO framework achieves a success rate of 66.24% and an average return of 3433.14 over 5000 evaluation episodes. Under the same original evaluation setting, PPO+ICM+Curriculum achieves a success rate of 68.62%, while its average return is 251.05 lower than that of CLI-PPO. The two configurations therefore produce different relationships between task completion and cumulative return. PPO+ICM+Curriculum completes a slightly larger proportion of the navigation episodes, whereas CLI-PPO accumulates a higher return under the specified reward function. Because the return combines distance, heading, goal, collision, and time-related terms, its higher value does not by itself indicate higher success-weighted path efficiency.

Figure 6 and Figure 7 report the in-distribution performance of the six trained policies under the original evaluation setting. The comparison shows how the tested configurations differ in their use of temporal representation, intrinsic exploration, and curriculum-based task scheduling. Variation across independent training runs is assessed under the same obstacle-density conditions.

**Figure 8:**
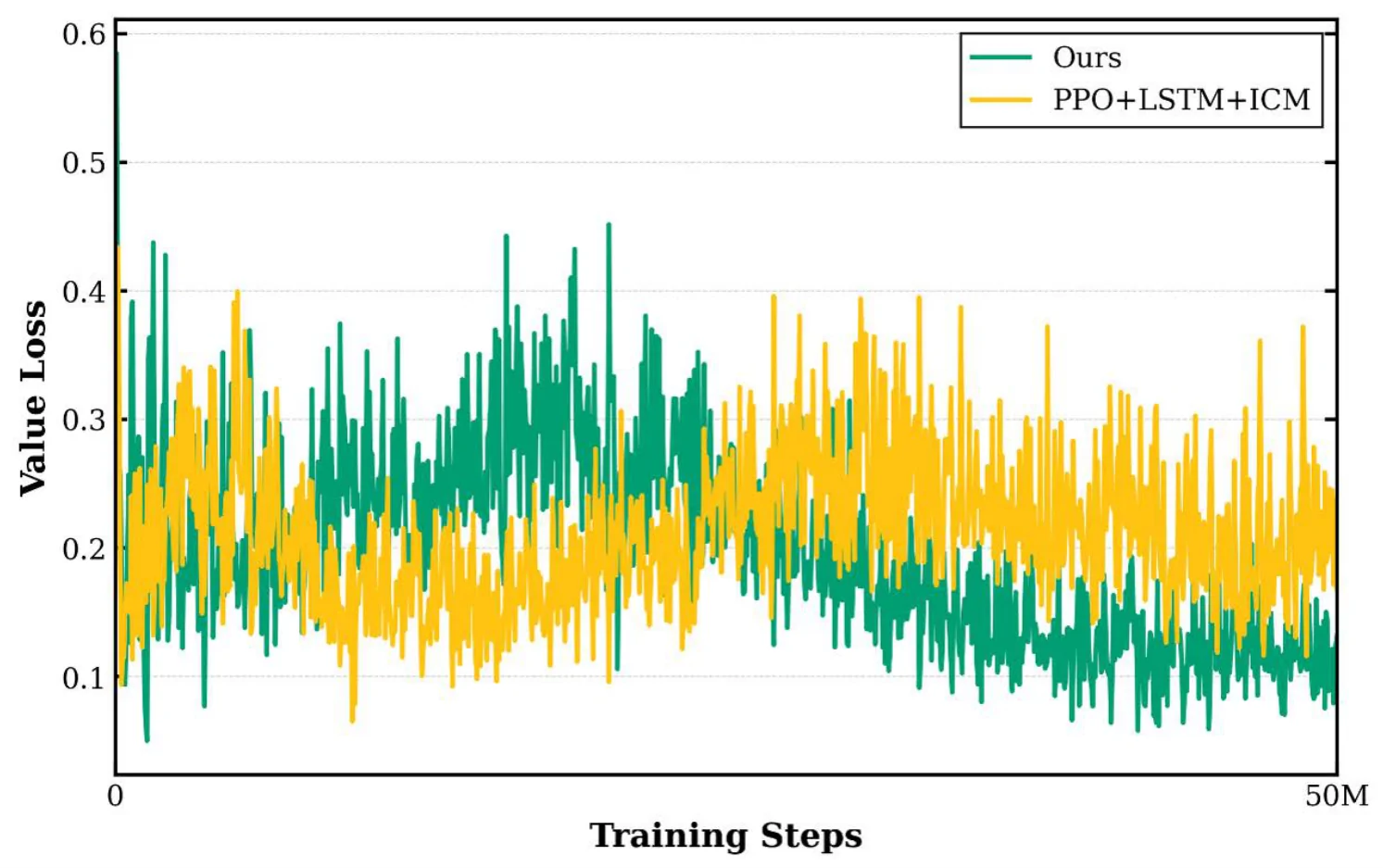
Comparison of Value Loss convergence curves between CLI-PPO and PPO+LSTM+ICM.

The impact of the curriculum learning mechanism on training stability is further investigated through the analysis of the Value Loss curves. As shown in Figure 8, the PPO+LSTM+ICM model without curriculum learning exhibits significant fluctuations in value loss throughout the entire training process. This indicates that directly optimizing a policy incorporating both temporal memory and exploration mechanisms in complex environments makes value function estimation highly susceptible to variations in environmental complexity and exploration behaviors.

In comparison, CLI-PPO also experiences certain fluctuations during the early training stage, which can be attributed to the initial process of establishing a complex policy under the curriculum learning framework. However, as the task difficulty gradually increases, the value loss of CLI-PPO decreases rapidly and maintains a relatively low fluctuation level. This demonstrates that curriculum learning effectively reduces the interference of complex tasks on the policy optimization process, enabling the value network to achieve more stable state-value estimation.

These results further verify the importance of curriculum learning in reinforcement learning training. By appropriately regulating task difficulty, curriculum learning improves policy convergence stability and facilitates more effective cooperation among multiple components within the integrated framework, ultimately enhancing the agent’s adaptability to complex environments.

#### 3.6.1. Simulation-Based Navigation Evaluation

**Figure 9:**
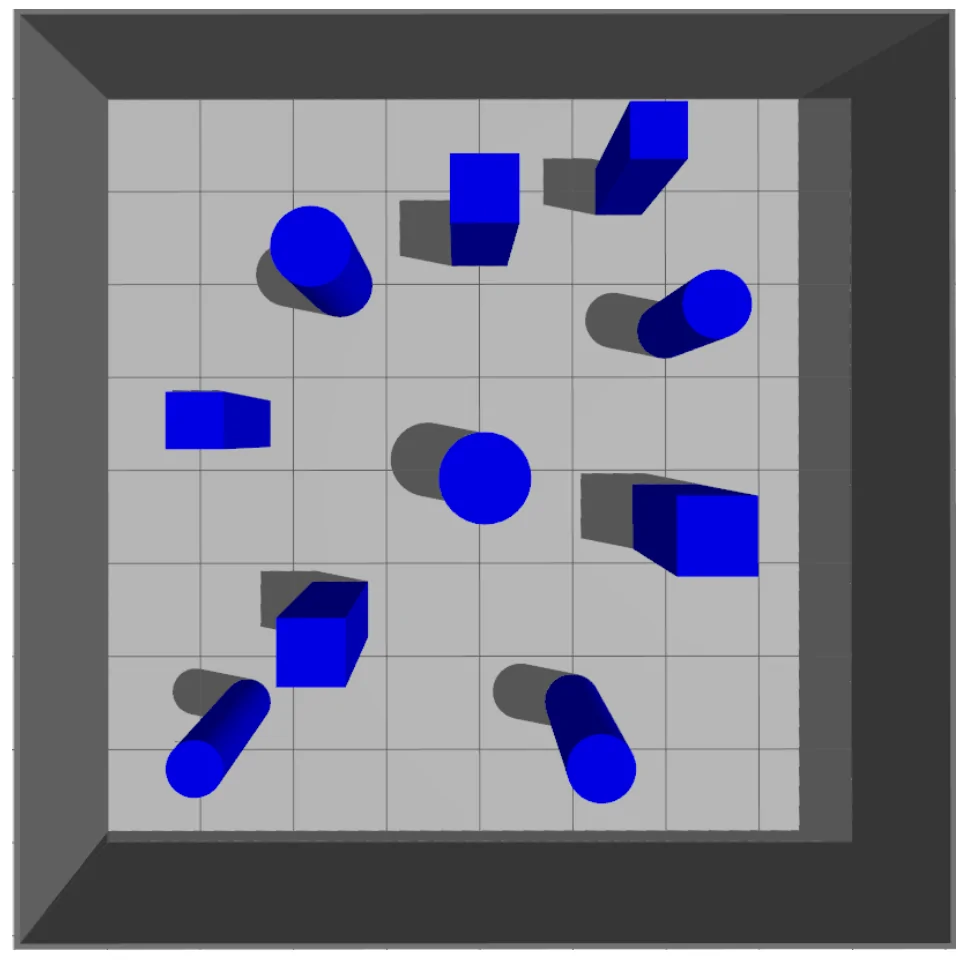
Illustration of the simulation environment used in this experiment. The number and size of obstacles are consistent with those in the training environment, ensuring that the testing conditions remain consistent with the training distribution.

Based on the experimental settings, PPO+ICM+Curriculum, which achieves a comparable success rate to CLI-PPO, is selected as the baseline for trajectory comparison to further evaluate the navigation performance of the proposed framework. Since both methods exhibit similar task completion capabilities, the influence of success rate differences can be minimized, allowing a more detailed investigation of the differences in path execution efficiency and motion behaviors between the two strategies. The simulation environment used for this trajectory evaluation is illustrated in Figure 9.

The trajectory comparison provides a qualitative view of the local motion patterns generated by the two policies under the simulation conditions shown in Figure 9. The associated navigation times and traveled path lengths are reported in Table 4 and Table 5.

**Figure 10:**
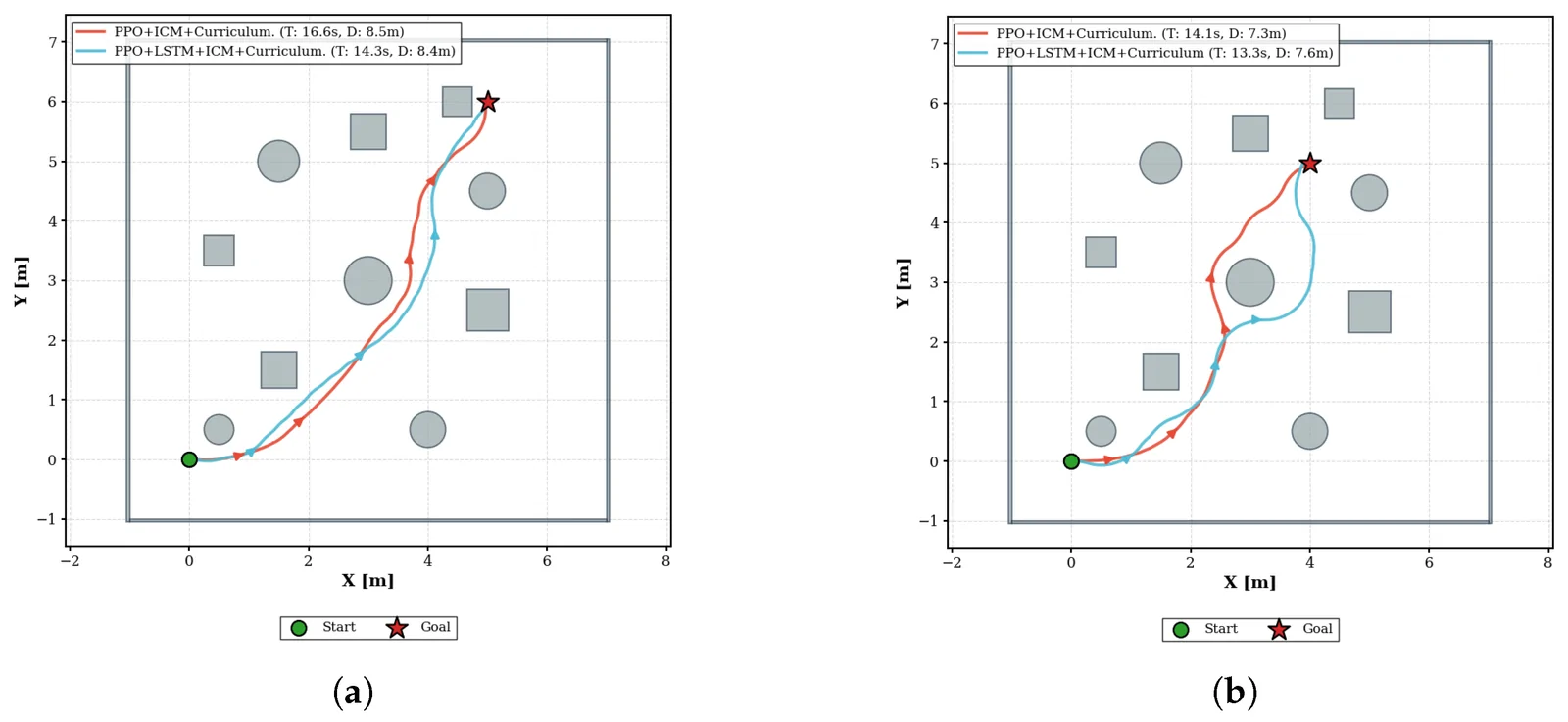
Comparison of typical navigation trajectories between CLI-PPO and PPO+ICM+Curriculum under the same testing conditions as the training environment. (**a**) Navigation trajectories for the first target point. (**b**) Navigation trajectories for the second target point.

In the experiment shown in Figure 10a, PPO+ICM+Curriculum required 16.6 s to complete the navigation task with a traveled path length of 8.5 m, whereas CLI-PPO required 14.3 s and traveled 8.4 m, as reported in Table 4. CLI-PPO therefore reduced the completion time by 2.3 s, corresponding to 13.9%, and reduced the traveled distance by 0.1 m. In the experiment shown in Figure 10b, both algorithms also successfully completed the navigation task. The completion time decreased from 14.1 s for PPO+ICM+Curriculum to 13.3 s for CLI-PPO, corresponding to a reduction of 5.7%, while the traveled path length increased from 7.3 m to 7.6 m, as reported in Table 5.

The two policies exhibit different local control patterns along the navigation paths. In PPO+ICM+Curriculum, intrinsic motivation guides exploration while curriculum learning progressively changes the distribution of start–goal configurations. CLI-PPO further incorporates recent observation history through the recurrent policy, and this temporal context is reflected in the sequence of directional adjustments made during obstacle avoidance.

The two trajectory plots and their corresponding measurements show successful task completion with different execution profiles. CLI-PPO reaches the target in a shorter time in both layouts, while the traveled distance differs between the two obstacle arrangements. The trajectory comparison provides a qualitative view of how temporal representation, intrinsic exploration, and curriculum-based task scheduling are reflected in local navigation behavior.

#### 3.6.2. Generalization Evaluation in Unseen Environments

Two selected distribution-shift scenarios were constructed to examine the navigation behavior of the trained policies when the obstacle geometry or obstacle number differs from the training environment. The first scenario changes the obstacle dimensions, while the second increases the obstacle number and introduces partial goal occlusion.

**Figure 11:**
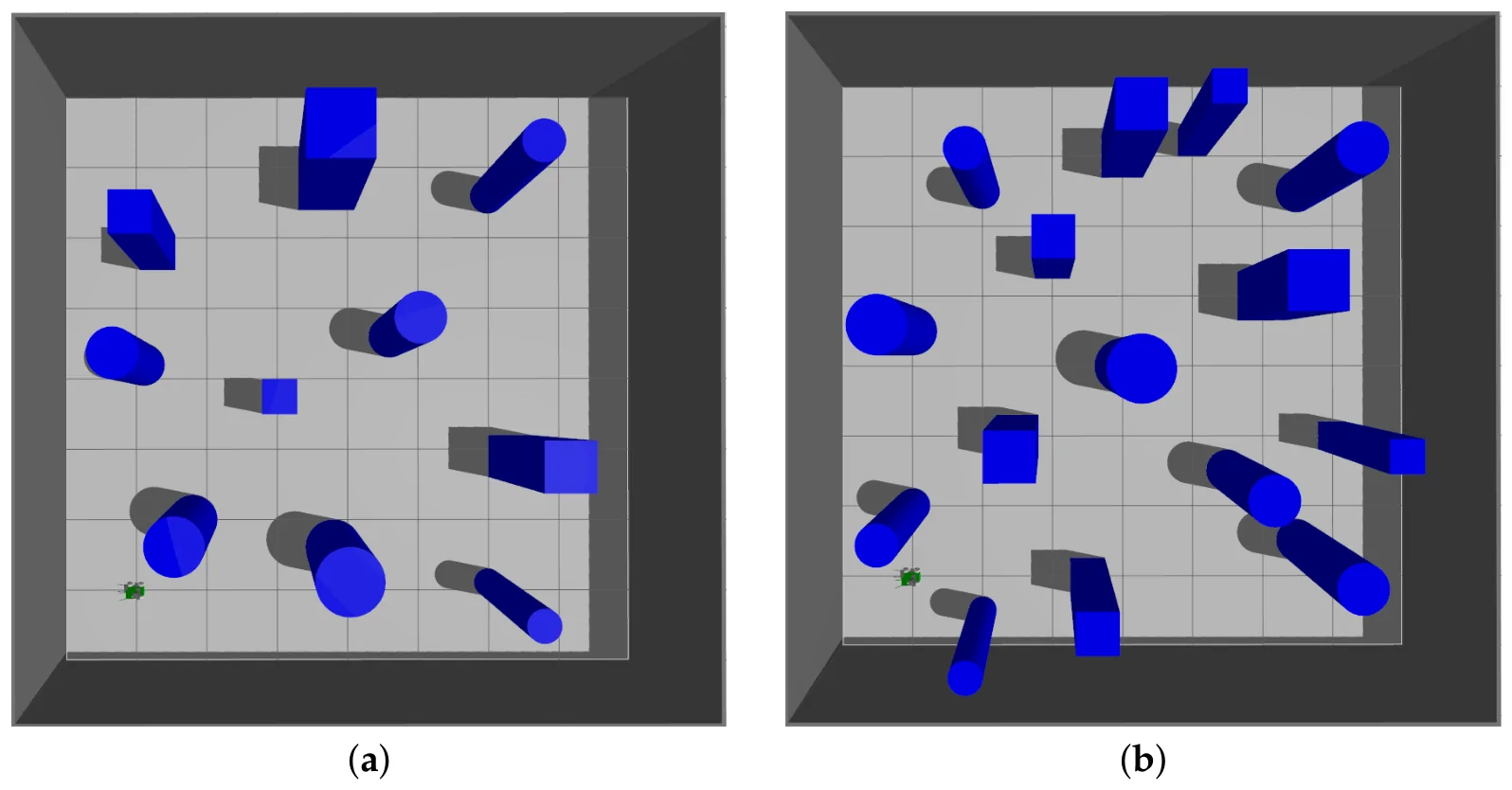
Two selected distribution-shift testing environments used to examine navigation behavior under obstacle-size variation and a 50% increase in obstacle number. (**a**) Obstacle-size variation scenario. (**b**) Increased-obstacle and partial-goal-occlusion scenario.

In the first scenario, the geometric dimensions of obstacles were different from those used during training, and the corresponding testing environment is illustrated in Figure 11a. The navigation outcomes, completion times, and traveled path lengths for this scenario are reported in Table 6.

In the second scenario, the number of obstacles was increased by 50% compared with the training environment, and the corresponding testing environment is shown in Figure 11b. The navigation outcomes, completion times, and traveled path lengths for this scenario are reported in Table 7.

**Figure 12:**
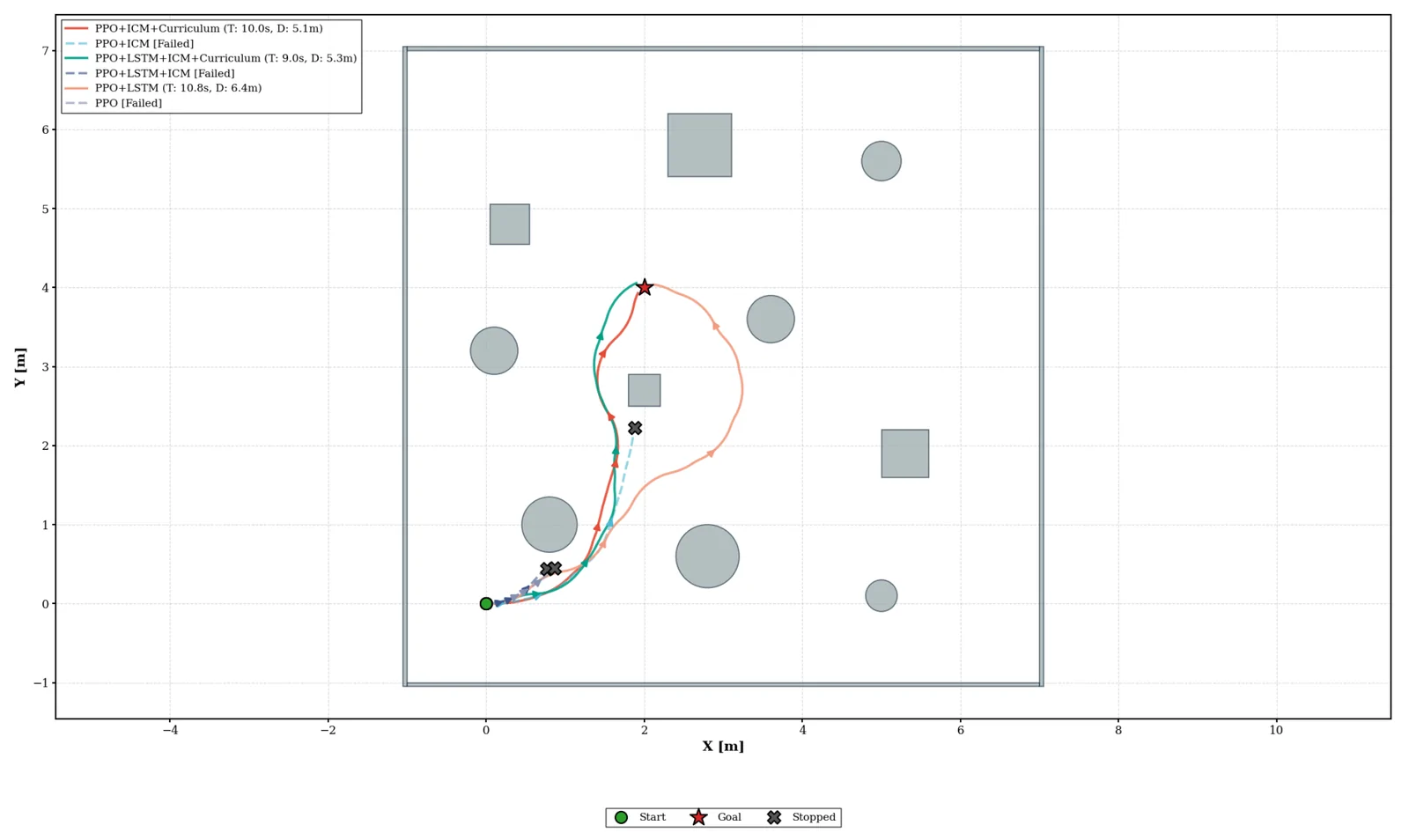
Comparison of navigation trajectories among different algorithms under the obstacle size distribution shift scenario shown in Figure 11a. The goal point is located in an open region, and the agent starts with a fixed rightward heading while performing path selection under limited perceptual information.

For the scenario shown in Figure 11a, the target point is placed in a relatively open region, while the robot is initialized with a fixed heading orientation toward the right side. Under the constraint of limited perceptual information, the robot is required to autonomously select an appropriate navigation path. The corresponding trajectory results are presented in Figure 12.

**Figure 13:**
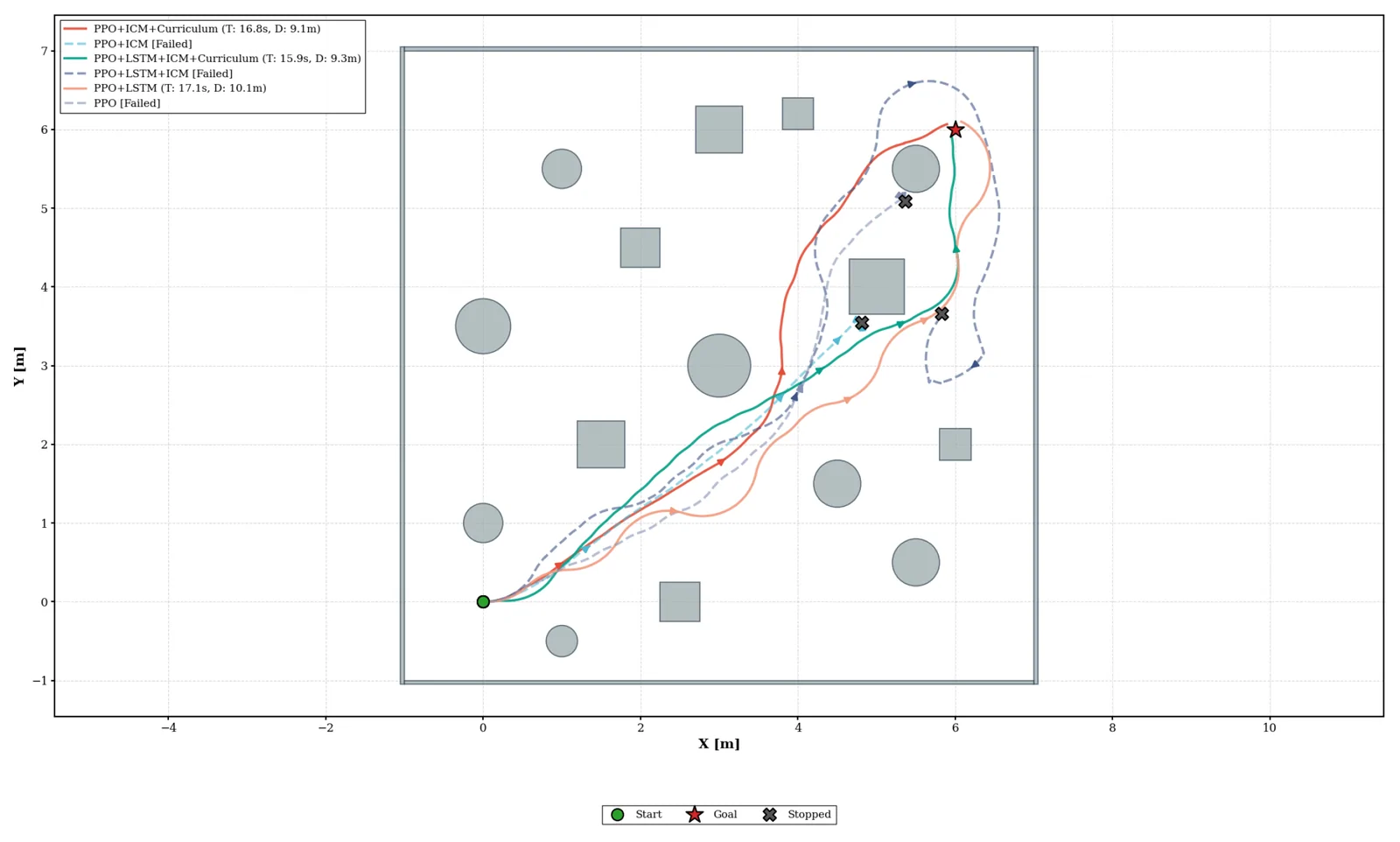
Comparison of typical navigation trajectories among different algorithms in the 50% increased obstacle number scenario shown in Figure 11b. The target is partially occluded by a cylindrical obstacle, and the agent is initialized with a fixed rightward heading under dense obstacle distribution.

For the scenario shown in Figure 11b, the target point is deliberately positioned behind a cylindrical obstacle to introduce severe goal occlusion. Under the same initial configuration, the robot is initialized with a fixed heading toward the right side, requiring the policy to discover feasible paths through regions with substantial obstacle-induced perceptual blockage. The corresponding trajectory comparisons are illustrated in Figure 13.

The two distribution-shift scenarios produce different navigation outcomes among the tested configurations. In the obstacle-size variation scenario shown in Figure 11a, PPO+LSTM, PPO+ICM+Curriculum, and CLI-PPO reach the target, whereas PPO, PPO+LSTM+ICM, and PPO+ICM do not complete the task. In the increased-obstacle scenario with partial goal occlusion shown in Figure 11b, the same three policies reach the target, while PPO, PPO+LSTM+ICM, and PPO+ICM fail to do so. PPO+LSTM completes these two scenarios in 10.8 s and 17.1 s, respectively. The corresponding navigation outcomes, completion times, and path lengths are reported in Table 6 and Table 7.

PPO+LSTM+ICM does not reach the target in either scenario, whereas PPO+ICM+Curriculum completes both tasks. During training, the latter configuration progressively expands the start–goal sampling regions as the navigation policy develops. Under the two reported distribution-shift conditions, this configuration reaches the target in both scenarios, while the configuration without curriculum does not. These outcomes describe the navigation behavior observed for the two specified obstacle arrangements.

CLI-PPO also completes both scenarios. In the obstacle-size variation scenario, it reaches the target in 9.0 s with a path length of 5.3 m, compared with 10.0 s and 5.1 m for PPO+ICM+Curriculum. In the increased-obstacle and partial-goal-occlusion scenario, CLI-PPO completes the task in 15.9 s with a path length of 9.3 m, while PPO+ICM+Curriculum requires 16.8 s and travels 9.1 m. The two scenarios therefore show differences in completion time and traveled distance among the policies that successfully reach the target.

The selected scenario results are followed by repeated density evaluations in environments containing 20 and 25 randomly distributed obstacles. These evaluations provide episode-level comparisons across the six configurations.The results for the six PPO configurations are summarized in Table 8 and Table 9, which report the success, collision, timeout, and mean cumulative reward under the two obstacle-density conditions.

Table 8 and Table 9 show that navigation performance decreases as the obstacle number increases from 20 to 25. Among the six evaluated configurations, PPO+ICM+Curriculum achieves the highest success rate in both environments, with values of 39.24% and 34.02%, respectively, and also gives the lowest collision rates of 60.76% and 65.96%. The complete PPO+LSTM+ICM+Curriculum configuration obtains success rates of 37.40% and 25.14%, while its collision rate increases from 61.82% to 74.18%. Its mean cumulative reward also decreases from 1278.51 to 594.18 as the obstacle density increases. These results show that the combination of intrinsic exploration and curriculum-based task progression maintains the most favorable task-completion performance among the tested configurations under the denser environments.

The preceding tables summarize the six-configuration density evaluation at 20 and 25 obstacles. A separate repeated-training summary across 10, 15, 20, and 25 obstacles is provided in Table 10, where the PPO-family results are aggregated across training seeds 43, 44, and 2026 and the SAC results are reported for the evaluated policy.

Table 10 summarizes the repeated-training results under four obstacle-density conditions. At 10 obstacles, CLI-PPO achieves a success rate of 62.72±3.59% and an average return of 3337.1±48.9, while PPO+ICM+Curriculum achieves a success rate of 63.83±6.61% and an Euclidean-reference SPL of 0.552±0.050. As the obstacle density increases, the success rate decreases for all policies. At 15, 20, and 25 obstacles, PPO+ICM+Curriculum achieves success rates of 46.59%, 33.44%, and 22.80%, compared with 45.38%, 30.52%, and 19.98% for CLI-PPO. The corresponding Euclidean-reference SPL values are 0.406, 0.292, and 0.202 for PPO+ICM+Curriculum, compared with 0.391, 0.265, and 0.175 for CLI-PPO.

CLI-PPO records higher mean returns at all four density levels, with values of 3337.1, 2300.8, 1458.0, and 847.4. Its success-rate standard deviations are also smaller than those of PPO+ICM+Curriculum at the corresponding densities. Success rate measures task completion, SPL combines successful completion with route efficiency, whereas cumulative return also reflects the numerical weighting of the distance, heading, goal, collision, and time terms in the reward function. The higher return of CLI-PPO therefore represents a different reward-accumulation pattern from the higher success rate and SPL obtained by PPO+ICM+Curriculum.

PPO+LSTM records accumulated turning per unit path of 1.322±0.039, 1.326±0.035, 1.331±0.031, and 1.332±0.028 rad/m at 10, 15, 20, and 25 obstacles, respectively, whereas the corresponding PPO values are 1.122±0.110, 0.977±0.017, 0.925±0.017, and 0.893±0.028 rad/m. The recurrent branch therefore changes the temporal representation used by the policy, while the measured trajectories contain more accumulated turning per unit path under the tested density conditions.

#### 3.6.3. Robustness to Normalized Observation and Control Perturbations

The density-generalization experiments evaluate the behavior of the policies under changes in obstacle number. To further examine the sensitivity of the learned policies to imperfect observations and delayed commands, an additional zero-shot perturbation evaluation was conducted using the frozen PPO and CLI-PPO policies.

All evaluations used 10 obstacles, 18 pooled LiDAR measurements, randomization_freq = 1, a minimum start–goal distance of 3 m, and a maximum episode length of 5000 environment steps. Each condition contained 5000 episodes, and the evaluation seed followed the same rule, $s_{i}=42+i$, where *i* denotes the episode index. The external reward configuration, network architecture, deployment settings, and environment parameters were kept unchanged. The perturbations were applied only during policy evaluation. LiDAR noise, LiDAR dropout, and velocity-channel noise were introduced in the normalized observation space. The LiDAR perturbations affected the 18 pooled LiDAR channels, while velocity noise was applied to the velocity channels of the observation vector. The action-delay condition inserted a delay of one environment control step between the predicted and executed commands.

**Table 10:** Navigation performance under different obstacle-density conditions. PPO-family entries are reported as the mean ± sample standard deviation across training seeds 43, 44, and 2026. SAC entries represent episode-level means from one evaluated SAC policy over 5000 episodes at each obstacle-density level.

Obst.	Method	Success (%)	Collision (%)	Timeout (%)	Return	Path (m)	NPL	SPL_E_	Clearance (m)	Smoothness (rad/m)
10	PPO	53.55±4.78	46.42±4.79	0.03±0.01	2423.3±199.6	3.650±0.142	0.798±0.030	0.480±0.045	0.340±0.013	1.122±0.110
10	PPO+LSTM	46.44±19.54	47.73±13.34	5.83±6.53	2913.0±490.2	6.117±1.923	1.351±0.448	0.372±0.188	0.318±0.045	1.322±0.039
10	PPO+ICM+Curriculum	63.83±6.61	36.13±6.66	0.04±0.05	3025.3±286.3	4.317±0.349	0.939±0.078	0.552±0.050	0.356±0.013	1.123±0.022
10	CLI-PPO	62.72±3.59	36.42±2.45	0.86±1.20	3337.1±48.9	4.800±0.786	1.049±0.179	0.530±0.055	0.349±0.006	1.318±0.007
10	SAC	21.80	76.28	1.92	624.5	1.831	0.426	0.214	0.287	–
15	PPO	34.71±4.95	65.27±4.97	0.02±0.02	1297.6±153.9	2.913±0.141	0.639±0.031	0.315±0.045	0.262±0.009	0.977±0.017
15	PPO+LSTM	35.31±7.69	62.79±5.64	1.89±2.46	2003.7±162.8	4.437±1.274	0.973±0.288	0.292±0.080	0.257±0.011	1.326±0.035
15	PPO+ICM+Curriculum	46.59±7.72	53.38±7.77	0.03±0.05	1956.4±321.4	3.636±0.463	0.791±0.101	0.406±0.059	0.275±0.012	1.053±0.032
15	CLI-PPO	45.38±0.55	54.29±0.78	0.33±0.50	2300.8±81.4	3.819±0.478	0.832±0.106	0.391±0.013	0.272±0.002	1.321±0.014
15	SAC	12.90	85.30	1.80	213.1	1.441	0.333	0.127	0.238	–
20	PPO	22.67±4.24	77.32±4.25	0.01±0.01	574.8±87.3	2.414±0.135	0.532±0.030	0.207±0.039	0.227±0.006	0.925±0.017
20	PPO+LSTM	24.27±2.37	74.67±0.74	1.06±1.70	1281.7±60.1	3.523±1.081	0.773±0.240	0.203±0.032	0.228±0.000	1.331±0.031
20	PPO+ICM+Curriculum	33.44±8.86	66.55±8.88	0.01±0.02	1185.4±338.6	3.092±0.524	0.676±0.114	0.292±0.069	0.237±0.010	1.001±0.032
20	CLI-PPO	30.52±1.80	69.37±1.90	0.11±0.20	1458.0±99.0	3.090±0.364	0.678±0.085	0.265±0.011	0.232±0.001	1.318±0.010
20	SAC	8.24	90.06	1.70	0.9	1.186	0.274	0.081	0.219	–
25	PPO	14.19±2.79	85.81±2.79	0.00±0.00	92.5±24.4	1.978±0.094	0.442±0.020	0.131±0.026	0.212±0.003	0.893±0.028
25	PPO+LSTM	14.57±1.88	84.35±0.83	1.08±1.77	686.2±55.2	2.803±0.881	0.622±0.196	0.123±0.024	0.212±0.001	1.332±0.028
25	PPO+ICM+Curriculum	22.80±7.85	77.20±7.85	0.00±0.00	576.0±276.6	2.520±0.508	0.559±0.113	0.202±0.063	0.219±0.007	0.965±0.042
25	CLI-PPO	19.98±2.11	80.00±2.09	0.02±0.02	847.4±98.0	2.490±0.264	0.553±0.061	0.175±0.013	0.215±0.002	1.319±0.014
25	SAC	5.16	93.80	1.04	−110.1	0.992	0.230	0.051	0.208	–

Across all four perturbation conditions, the results are shown in Table 11, CLI-PPO obtained a higher success-rate estimate than PPO. The success-rate advantage ranged from 3.9 percentage points under velocity noise to 6.8 percentage points under LiDAR noise. The corresponding collision-rate differences were also consistent, with CLI-PPO reducing the collision rate by 4.4–7.6 percentage points relative to PPO. These differences were observed under the same obstacle count, evaluation horizon, randomization frequency, and perturbation level.

The timeout rate remained below 1% for CLI-PPO in all four conditions, whereas the PPO checkpoint produced almost no timeout terminations. This indicates that the higher CLI-PPO success rate was mainly associated with a lower collision frequency rather than with a substantial reduction in episode duration. CLI-PPO also produced slightly higher mean path lengths than PPO, ranging from 7.480 to 7.674 m compared with 6.980 to 7.146 m for PPO. Nevertheless, the corresponding SPL_Euclidean_ values of CLI-PPO remained higher in all four conditions, with values between 0.258 and 0.268, compared with 0.239–0.260 for PPO.

The minimum-clearance values of the two methods were relatively close. CLI-PPO achieved values between 0.311 and 0.317 m, while PPO achieved values between 0.302 and 0.316 m. Thus, the success-rate difference cannot be attributed solely to a larger average clearance. With the smoothness definition used in this study, the metric represents the accumulated turning angle per unit path length. CLI-PPO produced values between 0.932 and 0.946 rad/m, which were higher than the corresponding PPO values of 0.786–0.869 rad/m. This suggests that the robustness advantage of CLI-PPO in task completion and collision avoidance was accompanied by additional turning under the tested perturbations, rather than by a uniform improvement in every trajectory-quality metric.

Under the matched evaluation protocol, the unperturbed CLI-PPO policy achieves a success rate of 65.60±47.51%, while PPO achieves 48.04±49.97%. With action delay, LiDAR dropout, LiDAR noise, and velocity noise, the success rates of CLI-PPO decrease to 51.54±49.98%, 50.32±50.00%, 52.16±49.96%, and 52.74±49.93%, respectively. Relative to the matched unperturbed condition, these changes correspond to decreases of 14.06, 15.28, 13.44, and 12.86 percentage points. LiDAR dropout produces the largest decrease, whereas velocity noise produces the smallest decrease for CLI-PPO.

For PPO, the corresponding success rates under the four perturbations are 45.02±49.76%, 46.22±49.86%, 45.42±49.79%, and 48.84±49.99%. Action delay, LiDAR dropout, and LiDAR noise reduce the success rate relative to the unperturbed condition. Under velocity noise, the measured velocity components are perturbed before action selection, which changes the local command sequence and the resulting avoidance trajectory. Some episodes consequently follow a perturbed route that avoids a collision encountered in the corresponding unperturbed trajectory, producing a small increase of 0.80 percentage points in the measured success rate. The overlapping confidence intervals indicate that this difference remains within the episode-level variation observed in the evaluation.

#### 3.6.4. Analysis of Experimental Results

The original in-distribution evaluation gives a checkpoint-level comparison: CLI-PPO achieves a success rate of 66.24%, a collision rate of 33.58%, and an average cumulative reward of 3433.14, compared with 46.06%, 53.94%, and 2083.21 for PPO. The repeated-training results in Table 10 quantify variation across independently trained policies under the density evaluation protocol. At 10 obstacles, CLI-PPO achieves a success rate of 62.72±3.59% and an average return of 3337.1±48.9, while PPO achieves 53.55±4.78% success and an average return of 2423.3±199.6. The original evaluation and the repeated-training evaluation describe different aspects of the learned policies. The former gives the performance of one selected checkpoint, whereas the latter shows how the results vary across independently trained policies.

Table 10 shows that PPO+ICM+Curriculum achieves higher mean success rates and Euclidean-reference SPL than CLI-PPO at all four obstacle densities. At 10 obstacles, the two success rates are 63.83±6.61% and 62.72±3.59%, and the corresponding SPL values are 0.552±0.050 and 0.530±0.055, respectively. At 15, 20, and 25 obstacles, PPO+ICM+Curriculum achieves success rates of 46.59%, 33.44%, and 22.80%, compared with 45.38%, 30.52%, and 19.98% for CLI-PPO. Its SPL values at these densities are 0.406, 0.292, and 0.202, while the corresponding CLI-PPO values are 0.391, 0.265, and 0.175.

CLI-PPO obtains higher mean returns at 10, 15, 20, and 25 obstacles, with values of 3337.1, 2300.8, 1458.0, and 847.4. Its success-rate standard deviations are also smaller at the corresponding density levels. Success rate measures task completion, SPL combines successful completion with route efficiency, and cumulative return is determined by the numerical structure of the complete reward function. The higher return of CLI-PPO therefore occurs together with lower success rate and SPL in the density evaluation. The complete configuration produces a different relationship between temporal observation processing, cumulative return, and the measured navigation outcomes.

The two structured distribution-shift scenarios examine the navigation response to modified obstacle geometry and an increased number of obstacles. In the obstacle-size variation scenario, PPO+LSTM, PPO+ICM+Curriculum, and CLI-PPO successfully reached the target, whereas PPO, PPO+LSTM+ICM, and PPO+ICM did not. The corresponding navigation times of the successful policies were 10.8 s, 10.0 s, and 9.0 s, respectively. Under the 50% increase in obstacle number, the same three policies completed the task, with navigation times of 17.1 s, 16.8 s, and 15.9 s, respectively. Under these two selected environmental changes, CLI-PPO recorded the shortest completion time among the successful policies. These scenario results complement the density-wise evaluation by showing how the policies behaved in specific changed environments.

For the evaluated SAC policy, the success rate decreases from 21.80% to 5.16%, while the collision rate increases from 76.28% to 93.80% as the obstacle count increases from 10 to 25. The shorter path lengths occur together with frequent collision termination. These measurements describe the behavior of the evaluated SAC policy under the specified training and evaluation configuration.

The matched perturbation evaluation shows higher episode-level success rates and lower collision rates for CLI-PPO than for PPO under the four tested conditions. This difference is accompanied by longer paths and larger accumulated turning per unit path for CLI-PPO. The perturbation results therefore describe a task-completion difference together with changes in trajectory geometry under the specified sensing and control disturbances.

## 4. Discussion

The experiments show that the three components influence different stages of the navigation process. The original in-distribution evaluation and the repeated training runs describe overall policy performance and variation between runs, while the structured distribution-shift and perturbation tests examine how the learned behavior changes when obstacle geometry, density, sensing, or command execution is altered. Temporal memory mainly affects how successive observations are incorporated into motion decisions; ICM changes the transitions explored during training; and Curriculum Learning determines how task difficulty is introduced. These differences lead to distinct performance patterns in success rate, return, path efficiency, and collision avoidance across the tested conditions.

The recurrent policy processes a sequence of observations rather than an isolated measurement at each decision step. PPO+LSTM and CLI-PPO both reach the target in the two selected distribution-shift scenarios, although their completion times and local paths differ. Across the four obstacle-density conditions, PPO+LSTM does not maintain a higher success rate than PPO, and its accumulated turning per unit path is consistently larger. In the present setting, temporal information therefore changes the policy representation of observation history, while the resulting trajectories retain more directional variation under the accumulated-turning metric.

The original ablation results show that adding ICM changes the policy behavior under sparse external feedback. The prediction-based signal supplies additional information about transition dynamics during training, but it does not directly measure progress toward the target or obstacle clearance. The resulting navigation performance therefore depends on how the learned exploration behavior interacts with the external reward and the task distribution.

As obstacle density increases, PPO+ICM+Curriculum maintains higher success rates and Euclidean-reference SPL than CLI-PPO, with the difference becoming more visible at 20 and 25 obstacles. CLI-PPO produces higher cumulative returns and smaller observed success-rate deviations across the repeated training runs. Its shorter completion times in the two structured distribution-shift scenarios describe behavior in those selected environments. The density results and the trajectory examples therefore capture different aspects of the learned policies. The recurrent branch changes the use of observation history, while its effect on task completion and route efficiency depends on the evaluated condition.

For the evaluated SAC policy, the success rate decreases as the obstacle number increases, while collision frequency rises. The corresponding path lengths become shorter because many episodes terminate before the target is reached. Path length is therefore interpreted together with success, collision, timeout, SPL, clearance, and smoothness. These measurements describe the behavior of the evaluated SAC policy under the specified configuration and remain limited to this task-matched reference.

The matched perturbation evaluation shows higher episode-level success estimates and lower collision estimates for CLI-PPO than for PPO under the four tested conditions. These values are accompanied by longer paths and larger accumulated turning per unit path for CLI-PPO. The result describes the response of the two policies to the specified sensing and control disturbances, including a difference in task completion together with changes in trajectory geometry. It does not imply a uniform improvement in every motion-quality metric.

The design choices adopted in this study are closely related to the requirements of the navigation task, and several limitations remain within this scope. The ablations do not include a simpler recurrent structure such as GRU, a capacity-matched recurrent comparison, or alternative module orders. The curriculum threshold, success window, and cooldown were selected empirically according to the task difficulty and the observed training behavior, so they represent task-specific settings rather than generally optimized values. The ICM branch uses prediction error as a general transition-novelty signal instead of adding hand-designed terms for target progress or obstacle clearance. This keeps the exploration signal separate from the task reward, but it also means that the usefulness and safety of an unfamiliar transition are not evaluated explicitly. The SAC evaluation is based on one trained policy under the common task configuration. Independent SAC training seeds and a separate algorithm-specific hyperparameter study were not included, so the reported values characterize this evaluated policy under the specified conditions.

The current validation is simulation-based and uses static obstacles. The robustness experiments cover the specified normalized observation and control disturbances, while dynamic obstacles, localization uncertainty, actuator limits, and physical robot constraints remain outside the present evaluation. Future work will examine task-aware intrinsic rewards, adaptive curriculum schedules, capacity-matched recurrent policies, dynamic environments, and physical robot validation.

Overall, the results show a conditional division of performance across the tested navigation conditions. PPO+ICM+Curriculum maintains higher task-completion rates and Euclidean-reference SPL as obstacle density increases, whereas CLI-PPO maintains higher returns, lower success-rate variation across the training seeds, shorter completion times in the selected structured distribution-shift scenarios, and higher success with lower collision rates under the tested observation and control perturbations. LSTM contributes temporal context, ICM changes the exploration signal, and Curriculum Learning controls the progression of task difficulty. Their combination produces different advantages across navigation conditions rather than the same improvement in every environment and metric.

## 5. Conclusions

This work presents CLI-PPO, a navigation framework that integrates temporal observation processing, action-conditioned curiosity, and progressive task training for unknown multi-obstacle environments. The hybrid reward function combines target guidance, obstacle avoidance, and exploration-related feedback during policy learning.

The experiments show that temporal representation, intrinsic exploration, and curriculum-based task scheduling affect different aspects of mapless navigation. PPO+ICM+ Curriculum achieves higher mean success rates and Euclidean-reference SPL across the tested obstacle-density conditions. CLI-PPO obtains higher cumulative returns, smaller observed success-rate deviations across the repeated training runs, and shorter completion times in the two selected structured distribution-shift scenarios. The recurrent branch therefore changes the behavior of the integrated policy, while its effect on task completion and route efficiency depends on the evaluation condition. The results show different performance characteristics across task completion, route efficiency, cumulative return, and response to the tested environmental changes.

The PPO-family experiments use repeated training seeds, whereas the SAC results describe one evaluated policy under the corresponding task definition, reward formulation, obstacle-generation procedure, and evaluation protocol. The reported measurements therefore characterize the tested policies under their specified simulation conditions. This setting allows the effects of temporal representation, intrinsic exploration, and curriculum-based training to be examined within the same navigation task. The normalized perturbation tests further assessed the response of the learned policies to controlled sensing and command disturbances, while the full variability of physical sensor measurements and robot execution was not included in the present evaluation. Future work will transfer the framework to real LiDAR, odometry, and inertial measurements and examine sensor noise, localization uncertainty, actuation delay, and dynamic obstacles on a physical robot.

Future work will prioritize the physical deployment of CLI-PPO. The framework will be integrated with real LiDAR, odometry, and inertial measurements and combined with a low-level safety controller to constrain unsafe policy commands during execution. The adapted system will then be transferred to a physical robot for navigation in environments involving sensing uncertainty, actuation delay, and dynamic obstacles, with emphasis on safe, reliable, and efficient operation.

## Figures and Tables

**Table 1:** Navigation performance of CLI-PPO policies trained with different intrinsic-reward coefficients β. Success rate is reported as the episode-level mean with the 95% confidence interval in parentheses. All remaining metrics are reported as episode-level means.

ICM β	Success	Collision	Timeout	Return	Steps	Path Length (m)	Normalized Path	SPL_Euclidean_	Min. Clearance (m)	Smoothness (rad/m)
0.05	54.8% (53.4–56.2%)	44.4%	0.8%	2850.23	1113.90	4.248	0.933	0.460	0.338	1.286
0.10	65.6% (64.3–66.9%)	34.2%	0.2%	3384.57	1120.34	4.437	0.969	0.565	0.353	1.316
0.20	63.8% (62.5–65.1%)	36.0%	0.1%	3286.03	1077.51	4.259	0.924	0.558	0.352	1.326

**Table 2:** Summary of reward function coefficients, derived reward terms, and associated state variables.

Symbol	Value	Description
$r_{d}$	400	Coefficient for distance improvement reward
$r_{h}$	300	Coefficient for heading improvement reward
$r_{c}$	−250	Penalty coefficient for collision
$I_{goal}$	{0,1}	Binary indicator of goal achievement
$\Delta d_{approach}$	$\max(d_{t-1}-d_{t},\;0)$	Distance reduction toward the goal
$\Delta h_{approach}$	$\max(∥\psi_{t-1}∥-∥\psi_{t}∥,\;0)$	Heading deviation reduction
$\psi_{t-1}$	−−−	Heading deviation at previous time step
$\psi_{t}$	−−−	Heading deviation at current time step
$r_{g}$	3000	Sparse reward coefficient for goal achievement
$I_{collision}$	{0,1}	Binary indicator of collision occurrence
$r_{t}$	−0.5	Fixed per-step time penalty

**Table 3:** Hyperparameter settings of the algorithm.

Hyperparameter	Value
Number of parallel environments	24
Total timesteps	$5\times 10^{7}$
PPO update epochs	10
Discount factor	0.999
GAE parameter	0.95
Learning rate	$1\times 10^{-5}$
PPO clipping coefficient	0.2
Entropy coefficient	0.01
Value loss coefficient	1.0
Max gradient norm	0.5
SDE sample frequency	4
Intrinsic reward coefficient	0.1
Curriculum success threshold	0.7
Curriculum cooldown steps	100
Maximum episode length	3000

**Table 4:** Figure 10a. Comparison of traveled path length and navigation time among different algorithms.

Algorithm	Navigation Time (s)	Path Length (m)	Result
PPO+ICM+Curriculum	16.6	8.5	Succeed
CLI-PPO	14.3	8.4	Succeed

**Table 5:** Figure 10b. Comparison of navigation efficiency in terms of traveled distance and completion time between CLI-PPO and PPO+ICM+Curriculum.

Algorithm	Navigation Time (s)	Path Length (m)	Result
PPO+ICM+CURRICULUM	14.1	7.3	Succeed
CLI-PPO	13.3	7.6	Succeed

**Table 6:** Scenario-specific navigation outcomes under the obstacle-size distribution shift.

Algorithm	Navigation Time (s)	Path Length (m)	Result
PPO	–	–	Failed
PPO+LSTM	10.8	6.4	Succeed
PPO+LSTM+ICM	–	–	Failed
PPO+ICM	–	–	Failed
PPO+ICM+CURRICULUM	10.0	5.1	Succeed
CLI-PPO	9.0	5.3	Succeed

**Table 7:** Scenario-specific navigation outcomes under goal occlusion and a 50% increase in obstacle number relative to the training environment.

Algorithm	Navigation Time (s)	Path Length (m)	Result
PPO	–	–	Failed
PPO+LSTM	17.1	10.1	Succeed
PPO+LSTM+ICM	–	–	Failed
PPO+ICM	–	–	Failed
PPO+ICM+CURRICULUM	16.8	9.1	Succeed
CLI-PPO	15.9	9.3	Succeed

**Table 8:** Evaluation results in the 20-obstacle environment, corresponding to a 100% increase in obstacle number relative to the 10-obstacle training condition.

Algorithm	Success (%)	Collision (%)	Timeout (%)	Mean Cumulative Reward
PPO+LSTM+ICM+Curriculum	37.40	61.82	0.78	1278.51
PPO+ICM+Curriculum	39.24	60.76	0.00	1338.59
PPO+ICM	24.84	75.04	0.12	475.05
PPO+LSTM+ICM	28.86	67.94	3.20	1154.27
PPO+LSTM	24.88	74.84	0.28	1249.80
PPO	17.80	82.20	0.00	483.86

**Table 9:** Evaluation results in the 25-obstacle environment, corresponding to a 150% increase in obstacle number relative to the 10-obstacle training condition.

Algorithm	Success (%)	Collision (%)	Timeout (%)	Mean Cumulative Reward
PPO+LSTM+ICM+Curriculum	25.14	74.18	0.68	594.18
PPO+ICM+Curriculum	34.02	65.96	0.02	982.71
PPO+ICM	12.78	87.18	0.04	−214.41
PPO+LSTM+ICM	17.06	77.70	5.24	367.30
PPO+LSTM	16.98	83.02	0.00	740.90
PPO	9.94	90.06	0.00	43.63

**Table 11:** Robustness evaluation of PPO and CLI-PPO under matched normalized observation and control perturbation protocols. The table includes an unperturbed reference condition evaluated with the same episode horizon, start–goal constraint, obstacle configuration, and randomization rule as the perturbed conditions. Success rate is reported as the episode-level mean ± standard deviation, followed by the 95% confidence interval in parentheses. All remaining values are episode-level means.

Method	Perturbation	Level	Success	Collision	Timeout	Steps	Path Length (m)	SPL_Euclidean_	Min. Clearance (m)	Smoothness (rad/m)
CLI-PPO	None	0	65.60 ± 47.51% (64.27–66.90%)	34.22%	1.80%	1120.05	4.436	0.565	0.353	1.316
CLI-PPO	Action delay	1 env. step	51.54 ± 49.98% (50.15–52.93%)	47.6%	0.8%	1087.07	7.629	0.261	0.312	0.945
CLI-PPO	LiDAR dropout	0.10	50.32 ± 50.00% (48.93–51.71%)	49.4%	0.3%	1014.82	7.480	0.258	0.311	0.932
CLI-PPO	LiDAR noise	0.03	52.16 ± 49.96% (50.78–53.54%)	47.0%	0.8%	1093.14	7.665	0.264	0.316	0.946
CLI-PPO	Velocity noise	0.03	52.74 ± 49.93% (51.36–54.12%)	46.4%	0.8%	1092.81	7.674	0.268	0.317	0.944
PPO	None	0	48.04 ± 49.97% (46.66–49.43%)	51.94%	0.02%	942.27	3.512	0.428	0.326	1.053
PPO	Action delay	1 env. step	45.02 ± 49.76% (43.64–46.40%)	55.0%	0.0%	922.54	7.021	0.239	0.306	0.869
PPO	LiDAR dropout	0.10	46.22 ± 49.86% (44.84–47.60%)	53.8%	0.0%	905.67	7.001	0.246	0.311	0.786
PPO	LiDAR noise	0.03	45.42 ± 49.79% (44.04–46.80%)	54.6%	0.0%	915.48	6.980	0.243	0.302	0.805
PPO	Velocity noise	0.03	48.84 ± 49.99% (47.45–50.23%)	51.1%	0.0%	949.58	7.146	0.260	0.316	0.818

## Data Availability

The original contributions presented in this study are included in the article. Further inquiries can be directed to the corresponding author.
